# Natural Killer Cell‐Mediated Antitumor Immunity: Molecular Mechanisms and Clinical Applications

**DOI:** 10.1002/mco2.70387

**Published:** 2025-09-14

**Authors:** Nanzhi Luo, Cong Chen, Wenjing Zhou, Jianqi Hao, Song He, Yu Liu, Yin Ku, Linhua Huang, Chuanfen Zhang, Yueli Shu, Xiaoqing Wu, Yaojia Zhou, Jian Zhang

**Affiliations:** ^1^ Department of Thoracic Surgery and Institute of Thoracic Oncology Frontiers Science Center For Disease‐Related Molecular Network West China Hospital of Sichuan University Chengdu China; ^2^ Faculty of Dentistry The University of Hong Kong Hong Kong China; ^3^ Department of Anesthesiology Southwest Medical University Luzhou China; ^4^ Animal Experimental Center of West China Hospital Chengdu China

**Keywords:** clinical strategies, metabolic reprogramming, molecular crosstalk, NK cells, TME

## Abstract

Natural killer (NK) cells are pivotal effectors in innate antitumor immunity by mediating cytotoxicity, secreting cytokines, or expressing cell membrane receptors, which facilitate interactions with other immune cells. The cytotoxic activity and immune function of NK cells are governed by dynamic receptor–ligand interactions, cytokine networks, and metabolic–epigenetic crosstalk within the tumor microenvironment (TME). Recent years, NK cell‐based therapies are emerging as a promising clinical approach for antitumor treatment, owing to their rapid response, unique recognition mechanisms, potent cytotoxic capabilities, and memory‐like characteristics, along with their low risk of posttreatment adverse effects and cost effectiveness. However, immunosuppression and metabolic reprogramming driven by TME subvert NK cell surveillance, impairing its antitumor function. This review comprehensively details molecular mechanisms underpinning NK cell dysfunction, including dysregulated activating/inhibitory receptor signaling, metabolic reprogramming, and epigenetic silencing of effector genes. We further synthesize advances in clinical strategies to restore NK cytotoxicity including ex vivo expansion for adoptive transfer, chimeric antigen receptor‐NK engineering, TME‐remodeling agents, immune checkpoint blockade, cytokine‐based therapies, and NK cell engagers targeting tumor antigens. By bridging mechanistic insights with translational applications, this work provides a framework for rationally designed NK cell‐based immunotherapies to overcome resistance across solid and hematologic malignancies.

## Graphical abstract

NK cells eliminate tumors via cytotoxicity and cytokine secretion. Tumor microenvironment‐driven immunosuppression and metabolic reprogramming subvert NK cells’ cytotoxicity. The current clinical strategies to restore NK activity include ex vivo expansion for adoptive transfer, CAR‐NK engineering, TME‐remodeling agents, immune checkpoint blockade, cytokine‐based therapies, and NK cell engagers targeting tumor antigens.

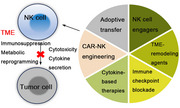

## 1 Introduction

Natural killer (NK) cells play a crucial role in innate immunity and are extensively distributed across various tissues and organs within the human body. Studies have revealed that NK cells executed immune functions by mediating cytotoxicity, secreting various cytokines, or expressing cell membrane receptors, which facilitate interactions with other immune cells [1].

The activation of NK cells can be oriented by multifaceted regulators. For example, epigenetic modifications including DNA methylation (such as killer cell immunoglobulin [Ig]‐like receptors [KIRs], interferon‐g [IFNG], and natural killer group 2A [NKG2A]) or histone modifications (such as AcH3, AcH4, and H3K4me3 in natural killer group 2D [NKG2D] gene) of transcriptional regions from specific genes play a crucial role in regulating the proliferation, differentiation, and functional activation of NK cells [2, 3, 4, 5]. Moreover, transcription factors such as signal transducer and activator of transcription (STAT) 4, STAT1, Zbtb32, and IRF8 also can directly impact NK cell activation. While epigenetic and transcriptional programs set the stage for NK cell activation, dynamic metabolic adaptations act as the executioner, translating environmental signals into functional outputs. Emerging evidence positions metabolic regulation as a central hub dictating NK cell fate and function. In noncancerous microenvironment, NK cells primarily rely on oxidative phosphorylation (OXPHOS) for energy homeostasis. Upon activation, they rapidly shift to aerobic glycolysis to meet the biosynthetic demands of cytokine production and cytotoxic granule synthesis [6]. Beyond glycolysis and OXPHOS, fatty acid oxidation (FAO) supports NK cell survival in nutrient‐poor environments, while glutamine (Gln) metabolism fuels rapid proliferation during activation.

Recent years, NK cell‐based therapies are emerging as a highly promising clinical approach for antitumor treatment, owing to their rapid response, unique recognition mechanisms, potent cytotoxic capabilities, and memory‐like (ML) characteristics, along with their low risk of posttreatment adverse effects and cost effectiveness [7, 8, 9, 10]. However, recent studies uncovered that the immune functions of NK cells were often suboptimal with the metabolic reprogramming in the tumor microenvironment (TME), which characterized by nutrient deprivation and accumulation of various tumor by‐products (e.g., lactic acid, hypoxia, adenosine [ADO]) with the activation of tumor‐related signaling pathways (such as the transforming growth factor‐β [TGF‐β] pathway, hypoxia–hypoxia‐inducible factor 1‐alpha [HIF‐1α] axis, and phosphoinositide 3‐kinase [PI3K]–protein kinase B [Akt] pathway), resulting in tumor progression or antitumor treatment failure. Thus, enhancing NK cell immune activity based on multidimensional molecular networks governing NK cells plasticity in TME to improve the efficacy of antitumor therapy are critical directions for both basic research and clinical applications in NK cell‐based therapies.

In this review, we integrated and discussed the receptor–ligand regulatory networks, metabolic reprogramming, epigenetic regulation, and associated molecular mechanisms underlying the altered immune functions of NK cells within the TME. Subsequently, based on an extensive review of the literature, we summarize current advances in therapeutic strategies—ranging from metabolic reprogramming agents and cytokine modulation to epigenetic interventions and adoptive NK cell transfer—that aim to restore or enhance NK cell antitumor activity. By bridging mechanistic insights with translational applications, our work offered a conceptual framework for designing more effective NK cell‐based cancer immunotherapies.

## 2 The Central Role of NK Cells in Antitumor Immunity

As the first line of defense in the innate immune system, NK cells differ from adaptive immune cells in that they exert direct effects on target cells without prior sensitization, allowing for a faster and broader response to antigens. NK cells are widely distributed throughout the body, particularly in peripheral blood, bone marrow, liver, and spleen. These characteristics underpin the crucial roles of NK cells in antitumor immunity, viral clearance, and autoimmune regulation. NK cells mediate antitumor immunity primarily through two mechanisms:

1. The cytotoxic activity of NK cells involves the release of lytic granules, such as perforin and granzymes, which induce tumor cell death [11]. NK cells release these lytic granules into the TME via exocytosis. Facilitated by integrins, the granules interact with tumor cells and enter through multiple pathways, such as pore formation in the membrane or endocytosis. Within tumor cells, they perform various functions, including the induction of single‐stranded DNA damage, direct proteolysis, and mitochondrial dysfunction, ultimately leading to apoptosis [12].
2. A wide spectrum of cytokines and chemokines—including IFN‐γ and tumor necrosis factor‐alpha (TNF‐α)—are secreted to facilitate interactions with other cells and activate coordinated immune responses. The activation of immune function of NK cells can be regulated by varies factors in TME. For example, upon external stimulation by cytokines such as interleukin (IL)‐2 or the combination of IL‐12 and IL‐15 in TME, NK cells in peripheral blood significantly upregulate their levels of IFN‐γ production [13]. Binding of IFN‐γ to its corresponding receptors on tumor cells initiates downstream signal transduction and orchestrates diverse immunological and cellular responses. A comprehensive discussion of these immunomodulatory processes is provided in Section 3 of this paper.

The functional contributions of NK cells to antitumor immunity differ across distinct NK cell subsets. Human NK cells are categorized into CD56^bright^CD16^−^ cells, which exhibit high CD56 and low CD16 expression, and CD56^dim^CD16^+^ cells, characterized by low CD56 and high CD16 expression, based on specific markers on their surface. Upon activation, CD56^bright^CD16^−^ NK cells exhibit enhanced cytokine secretion capabilities, whereas CD56^dim^CD16^+^ NK cells display superior cytotoxic killing abilities [14]. Additionally, it has been observed that memory NK cells residing in mouse liver tissue exhibit relatively low metabolic levels in the absence of external stimuli. However, upon re‐exposure to the same antigen, the metabolic rate of these memory NK cells increases dramatically, surpassing that of NK cells within the intrinsic immune system, thereby ensuring rapid proliferation and effective immune function [15]. Similarly, NK cells that have undergone the cell education process exhibit elevated levels of glucose transporter expression and glycolysis rates, thereby ensuring their mature function [16].

Leveraging both the two major immune strategies and the differentiation into distinct NK cell subsets, NK cells adapt to complex and fluctuating conditions, including both homeostatic condition and the TME. Meanwhile, various intrinsic and extrinsic factors—described in detail below—independently or cooperatively exert both stimulatory and inhibitory influences on NK cell function. In addition to exhibiting phenotypic and functional plasticity in response to hostile conditions, NK cells also fulfill an irreplaceable role in orchestrating effective antitumor immune responses.

## 3 Molecular Mechanisms Underlying NK Cell‐Mediated Antitumor Immunity

NK cell‐mediated antitumor immunity is a complex process shaped by the interplay of multiple factors, involving molecular mechanisms such as receptor–ligand interactions at the cell surface, cytokine‐driven signaling pathways, metabolic reprogramming, and epigenetic regulation. Subsequent sections will detail the molecular and cellular basis of NK cell crosstalk with other constituents of the TME, along with the positive and negative modulatory roles played by diverse regulatory factors.

### 3.1 Surface Receptor–Ligand Interactions in NK Cell Immune Regulation

A broad spectrum of receptors is expressed on the surface of NK cells, enabling them to engage with defined ligands on potential target cells and mediate direct immune effector functions. The functional outcome of NK cell–target cell interactions is determined by the balance between activating and inhibitory receptor signaling, resulting in either activation or suppression of NK cell activity. The transition between these states is tightly regulated by receptor–ligand interactions, enabling NK cells to fine‐tune their functional responses according to environmental cues, thereby preserving immune efficacy and avoiding autoreactivity [15]. Subsequent sections will explore how activating and inhibitory receptors, as well as their respective ligands, orchestrate the regulation of NK cell activity.

#### 3.1.1 Activating Receptors and Their Ligands

Tumor cell recognition and killing by NK cells are largely driven by a repertoire of activating receptors, notably including NKG2D, NK receptor group 2, member C (NKG2C), DNAX‐accessory molecule‐1 (DNAM‐1), CD244 (also known as 2B4), and the natural cytotoxicity receptors (NCRs) NKp30, NKp44, and NKp46 [15, 17]. The subsequent section will use prototypical activating receptors as examples to elucidate their roles in modulating NK cell‐mediated immune responses.

In humans, NKG2D primarily binds to eight stress‐induced ligands, including MHC class I chain‐related proteins A and B (MICA and MICB), as well as UL‐16 binding proteins (ULBPs), collectively referred to as NKG2D ligands (NKG2DLs). In mice, NKG2D interacts with ligands such as Rae‐1 (α–ε), MULT1, and H60 (a–c) [18]. Among all NKG2DLs, MICA and MICB were the earliest to be identified in humans and remain the most thoroughly investigated. Their expression is significantly upregulated on abnormal and tumor cells, thereby promoting NK cell‐mediated cytotoxicity. Clinical studies in colorectal cancer have demonstrated that high MICA expression is significantly associated with improved patient survival outcomes [19]. As tumors progress, tumor cells develop various strategies to reduce surface MICA/MICB expression and impair NKG2D–NKG2DL interactions, ultimately leading to immune escape [17, 20]. Studies have shown that tumor cells actively release MICA into the TME through proteolytic shedding and exosome‐mediated secretion, which in turn weakens NK cell‐mediated cytotoxic responses [21]. It is worth noting that soluble MICA has been proposed as a clinical screening marker, although further studies are required to confirm its applicability and diagnostic value across different populations. Similarly, in healthy wild‐type mice, NKG2D is normally activated, whereas tumor‐associated endothelial cells highly express RAE‐1ε, a ULBP family member, which triggers NKG2D internalization on NK cells, thereby impairing their immune function [22]. Additionally, high expression of ULBPs in patients with ovarian cancer has been associated with poor clinical outcomes [23]. Furthermore, multiple tumor types, including acute myeloid leukemia (AML) and glioma cells, have been shown to express a variety of NKG2DLs. As the disease progresses and the TME becomes increasingly complex, the expression profiles of these ligands undergo dynamic changes [20]. While NK cells with high NKG2D expression exert potent cytotoxic activity against tumor cells, NK cell subsets with high NKG2C expression typically emerge in response to viral infections and contribute to the formation of antigen‐specific human NK memory cells [24, 25].

DNAM‐1 is another critical activating receptor on NK cells that plays an essential role in tumor cell recognition and antitumor immune responses. The primary ligands for DNAM‐1 include the poliovirus receptor (PVR) and Nectin‐2 (CD112), both of which are upregulated on the surface of various tumor cells and in response to cellular stress [18, 26]. Similar to NKG2D, sustained engagement of DNAM‐1 with PVR on tumor cells leads to downregulation of DNAM‐1 on the NK cell surface, potentially through receptor internalization followed by proteasomal degradation or by shedding of PVR from the tumor cell membrane into the extracellular environment. As a result, DNAM‐1 surface levels decrease, its ligand binding is disrupted, and NK cell cytotoxicity against tumor cells is compromised. Since PVR is also recognized with high affinity by the inhibitory receptors CD96 and the immune checkpoint T cell immunoreceptor with Ig and ITIM domains (TIGIT), and Nectin‐2 is similarly bound by CD112R and TIGIT, the activating effects of DNAM‐1 on NK cells are determined by competitive binding with these receptors. In advanced stages of tumor progression, the upregulation of these inhibitory receptors suppresses DNAM‐1‐mediated activation of NK cells. Moreover, the involvement of these inhibitory receptors complicates the interactions between DNAM‐1 and other costimulatory receptors on NK cells. When multiple receptors are simultaneously engaged by their ligands, the activating receptors DNAM‐1, NKG2D, and CD244 can act synergistically to enhance signaling, thereby overcoming the immune evasion effects mediated by inhibitory receptors [27]. Furthermore, DNAM‐1 contributes critically to NK cell responses in tumors like melanoma that lack significant NKG2DL expression [28]. Conversely, other studies suggest that DNAM‐1‐mediated cytotoxicity can be impaired by NKG2D–ligand interactions that induce high TIGIT expression, and that DNAM‐1‐associated signaling pathways may be directly disrupted by NKG2D activation [29].

Although CD244 is commonly classified as an activating receptor on NK cells, accumulating evidence indicates that it exerts dual regulatory roles in NK cell‐mediated antitumor immunity [30]. The duality arises from the fact that both CD244 and its primary ligand, CD48, are broadly expressed across multiple types of immune cells. Within the complex TME, various immune cells—including CD4+ and CD8+ T cells as well as dendritic cells—interact with CD244‐expressing NK cells, exerting either synergistic or antagonistic effects that collectively shape NK cell immune responses through a balance of signals. It is also worth noting that the interaction between CD244 and CD48 can elicit opposing effects under different physiological or pathological contexts. In mice, disruption of CD244–CD48 binding impairs calcium signaling in NK cells, thereby compromising their cytotoxic activity and IFN‐γ production [31]. Conversely, in human hepatocellular carcinoma (HCC), CD48‐overexpressing monocytes within the TME engage CD244+ NK cells, leading to transient activation followed by rapid exhaustion and cell death [32]. Moreover, stimulation by CD48‐expressing tumor cells induces CD244 internalization on NK cells, reducing its surface expression and consequently impairing NK cell activation and cytotoxicity [33]. In addition to CD48, CD244 can also interact with the adaptor protein 3BP2 in humans, and this interaction has been shown to enhance NK cell cytotoxicity [34]. As more studies emerge, additional ligands of CD244 and receptor–ligand interactions are being identified, contributing to a more nuanced understanding of its dual regulatory role in NK cell biology.

NCRs, including NKp30, NKp44, and NKp46, were among the first identified activating receptors on NK cells. They mediate cytotoxicity and tumor cell lysis through interactions with their respective ligands, such as human leukocyte antigen (HLA)‐B‐associated transcript 3/Bcl‐2‐associated athanogene 6 (BAT3/BAG6), mixed‐lineage leukemia 5 (MLL5), and ecto‐calreticulin (ecto‐CRT) [35, 36]. Similar to CD244, NCRs are broadly expressed across multiple immune cells, which adds complexity to their roles in NK cell‐mediated immune responses. NK cells can express a single NCR or multiple NCRs simultaneously. By modulating the expression profiles of different NCRs, NK cells adapt to diverse microenvironments, exerting cytotoxic effects or producing cytokines to coordinate with other immune cells. The expression level of NCRs on the NK cell surface directly correlates with their cytotoxic potency against tumor cells. Moreover, NCRs can synergize with NKG2D, further enhancing NK cell‐mediated tumor clearance [36, 37].

With advances in NK cell research, increasing attention has been paid to the receptors shared between NK cells and other immune cells, as well as their functional interactions. The costimulatory receptor CD137 (also known as TNFRSF9 or 4‐1BB), which plays a critical role in CD8+ T cell biology, along with its monoclonal antibody (mAb) urelumab, has also attracted interest in the context of NK cell research. CD137 has been demonstrated to activate NK cells, enhance their cytotoxicity, and synergize with T cells in mediating antitumor responses [38, 39].

#### 3.1.2 Inhibitory Receptors and Immune Evasion Mechanisms

While activating receptors ensure the immediate effector function of NK cells, effective recognition and elimination of tumor cells also rely on the “education” of NK cells mediated by inhibitory receptors. Normal cells express self‐MHC class I molecules, which serve as ligands for NK cell inhibitory receptors and thereby prevent autoreactive immune responses. Through this educational process, NK cells become capable of mounting robust immune responses against tumor cells with downregulated MHC‐I expression. In contrast, NK cells lacking inhibitory receptor‐mediated stimulation typically exhibit diminished immune responses toward tumor cells [40]. Major inhibitory receptors include KIRs, NKG2A/CD94, and TIGIT [41]. The following section will discuss representative inhibitory receptors and their regulatory roles in modulating NK cell‐mediated immune responses.

The interaction between KIRs and their major ligands, particularly HLA‐C within the HLA class I family, is central to the education and functional calibration of NK cells. For instance, in patients with non‐Hodgkin lymphoma, a limited presence of KIR ligands promotes the expansion of “educated” KIR+ NK cells, which enhance the therapeutic efficacy of rituximab by facilitating tumor cell killing. However, in the presence of abundant KIR ligands within the TME, the immune system tends to generate poorly responsive KIR− NK cells, while KIR+ NK cells engage with HLA ligands, leading to suppression of their antitumor activity [42]. A similar phenomenon has been observed in non‐small cell lung cancer (NSCLC), where more than 50% of patients express KIR3DL1, and positivity for KIR2D is associated with significantly shorter overall survival compared with KIR2D‐negative patients [43]. Notably, B haplotypes of the KIR gene cluster can encode activating KIRs, whose ligands remain largely unidentified, and the functional consequences of their engagement on NK cell activity warrant further investigation [44, 45].

NKG2A, in complex with CD94, functions as an inhibitory receptor on NK cells [46]. The ligand for NKG2A, HLA‐E, is ubiquitously expressed on self‐cells in humans, allowing NKG2A‐expressing NK cells to maintain immune competence and functional readiness upon encountering self‐stimuli [44, 47]. However, in colorectal cancer, overexpression of NKG2A ligands by tumor cells can suppress NK cell‐mediated immunity, ultimately contributing to poor prognosis [48]. Similar observations have been reported across other tumor types, establishing NKG2A as an immune checkpoint receptor on NK cells [49]. Therapeutic strategies targeting NKG2A, including blocking antibodies and competitive inhibitors of its ligand on tumor cells, are currently under investigation and will be discussed in detail in Section 4 on clinical applications of NK cell‐based therapies.

TIGIT, a recently identified inhibitory immune checkpoint on NK cells, plays a crucial role in both antitumor immunity and NK cell education [40]. In wild‐type mice, the interaction between TIGIT on NK cells and its high‐affinity ligand PVR enhances NK cell effector function upon stimulation, whereas TIGIT+ NK cells from PVR‐deficient mice display impaired responses [40]. Similar to NKG2A, tumor cell‐expressed PVR binds TIGIT on NK cells, resulting in suppression of antitumor immunity. Consequently, therapeutic inhibitors targeting TIGIT or its interaction with PVR have attracted considerable research interest.

### 3.2 Cytokines and Signaling Pathways

**FIGURE 1:**
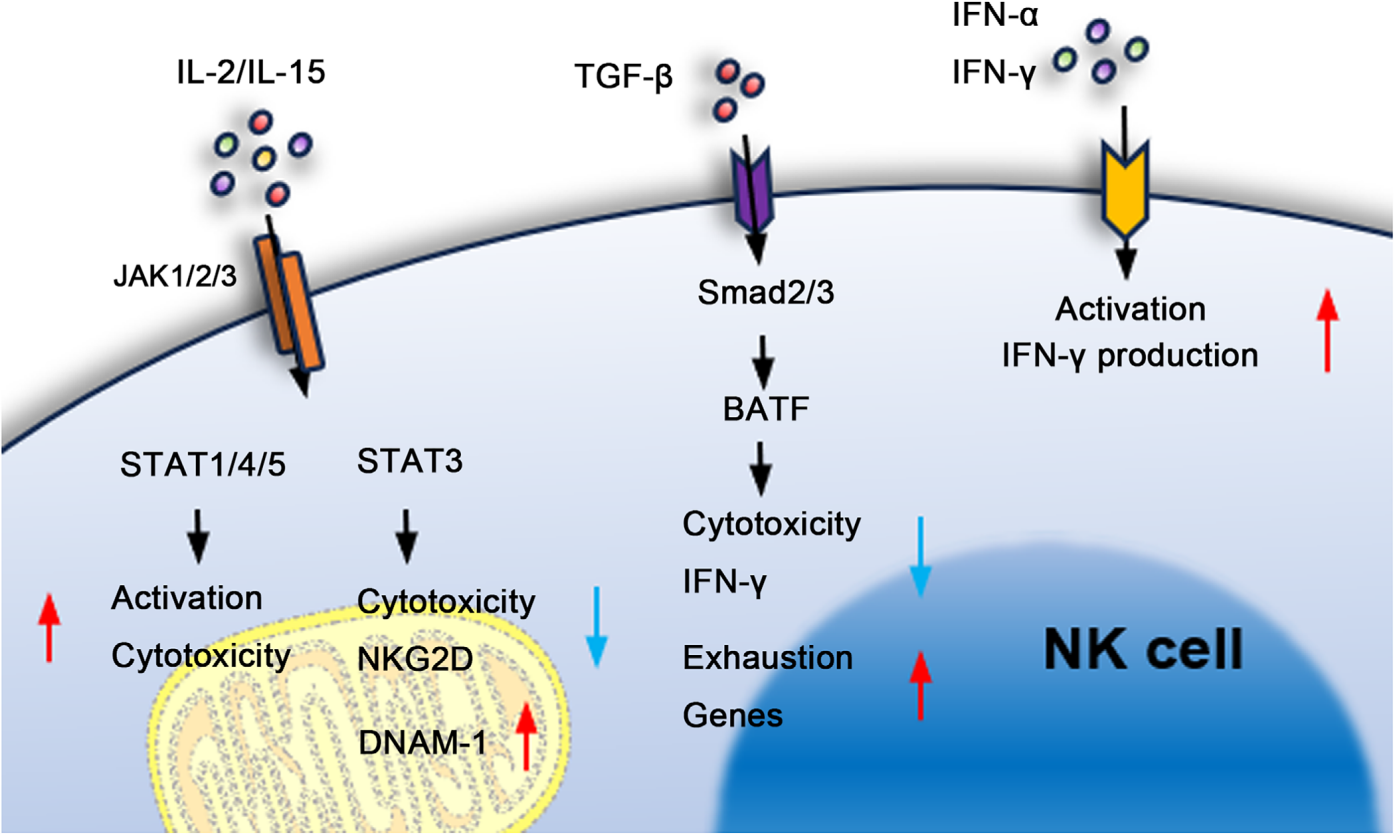
Cytokine‐ and signaling pathway‐mediated regulation of NK cell function within the TME. Multiple cytokines and checkpoint molecules—including IL‐2, IL‐12, IL‐15, and TGF‐β‐dynamically regulate NK cell activation, cytotoxicity, and survival through modulation of intracellular signaling cascades and cell–cell interactions within the TME. Engagement of IL‐2/IL‐15 receptors activates JAK1/2/3, leading to phosphorylation of STAT1/3/4/5. STAT1/4/5 promote NK cell proliferation, differentiation, and cytotoxicity, whereas STAT3 acts as a negative feedback regulator, suppressing NK cell tumor‐killing activity. Tumor‐derived TGF‐β activates Smad2/3 in NK cells, driving transcriptional programs—partly via BATF—that induce exhaustion, reduce IFN‐γ production, and promote epigenetic–metabolic reprogramming. NK cell‐derived IFN‐γ and IFN‐α act in autocrine/paracrine fashion to enhance NK cell differentiation into IFN‐γ‐producing subsets, amplifying antitumor immune responses despite proliferative suppression.

Researchers have confirmed that the status of NK cells can be regulated and impacted by various factors (cytokines IL‐2, IL‐12, IL‐15, TGF‐β, immune checkpoints, and metabolic checkpoints proteins such as mechanistic target of rapamycin complex 1 (mTORC1)) and exhibit dynamic fluctuation [21]. The regulatory effects of these factors on NK cells are exerted through the modulation of intracellular signaling cascades and the orchestration of cell–cell interactions between NK cells and other components of the TME. The underlying mechanisms are detailed below (Figure 1).

#### 3.2.1 IL‐2/IL‐15‐Driven JAK–STAT Signaling in NK Cell Activation

In the TME, the JAK–STAT signaling pathway mediated by cytokines such as IL‐2 and IL‐15 is critical for the proliferation, differentiation, and immune response of NK cells. Engagement of IL‐2/IL‐15 triggers JAK1/2/3 activation, leading to phosphorylation of STAT1/3/4/5, which translocate to the nucleus and regulate gene expression that governs NK cell activation and cytotoxicity [50]. Loss or inhibition of JAK1 or JAK3 reduces NK cell numbers and impairs their tumor recognition and cytotoxic functions [51, 52]. Similarly, deficiencies in downstream effectors STAT1, STAT4, or STAT5 result in impaired NK cell development, reduced cytotoxicity, and compromised cytokine secretion, with STAT5 acting as a key regulatory factor [50]. In contrast, STAT3 is not essential for NK cell development. Its phosphorylation acts as a negative feedback regulator within the JAK–STAT pathway, suppressing NK cell cytotoxicity against tumor cells. Interestingly, STAT3 deficiency enhances DNAM‐1 expression and NK cell cytotoxicity, while downregulating another activating receptor, NKG2D [53, 54]. These findings suggest a dual role for STAT3 in modulating NK cell function. Under the influence of multiple cytokines (e.g., IL‐2, IL‐15, IL‐10, IL‐21), STAT3 coordinates with other STAT family members to fine‐tune the JAK–STAT axis, where the dynamic interplay determines the magnitude of NK cell‐mediated immune responses.

#### 3.2.2 TGF‐β/Smad Signaling‐Driven Functional Impairment in NK Cells

Activation of the TGF‐β/Smad pathway in NK cells within the TME is a key driver of immune evasion by tumor cells. Studies on B‐acute lymphoblastic leukemia and AML have shown that TGF‐β in the TME activates this pathway, leading to reduced NK cell cytotoxicity, decreased IFN‐γ production, and the onset of NK cell exhaustion. In AML, the downstream effector BATF of Smad2/3 has been found to bind to exhaustion‐associated genes and contribute to epigenetic–metabolic reprogramming. Therefore, blockade of TGF‐β signaling may partially restore NK cell immune function and holds therapeutic potential in the treatment of leukemia [55, 56].

#### 3.2.3 IFN‐γ‐Mediated Positive Feedback Loop in NK Cell Function

NK cell activity is modulated by both external cues from the TME and autocrine/paracrine factors produced by NK cells themselves. Key among these are IFN‐γ and IFN‐α, which not only suppress NK cell proliferation but also reinforce immune activation. In particular, IFN‐α drives the differentiation of NK cells into IFN‐γ‐producing subsets, creating a positive feedback loop that amplifies their antitumor response [57, 58].

## 4 Metabolic Reprogramming

Once the immune responses are triggered, NK cells rely on the acquisition of large quantities of nutrients including glucose, amino acids, and fatty acids (FAs) from external sources. These nutrients serve a dual purpose for NK cells: on the one hand, they supply the raw materials necessary for the synthesis of biomolecules such as RNA, DNA, and proteins, which are crucial for cell survival and immune functions; on the other hand, they provide the substrates required for ATP synthesis in activated immune cells, sustaining the immune response. NK cells adapt to the environment by modulating their reliance on each metabolic pathway based on the distinct regulatory factors present in each setting.

During tumorigenesis and progression, due to the deprivation of resources and the discharge of a large amount of metabolic waste caused by the high metabolic state of the tumor, the surrounding microenvironment of the tumor becomes unsuitable for other cells to survive. NK cells have to develop metabolic reprogramming to better adapt to the TME. However, the metabolic reprogramming disrupts metabolic–function homeostasis, characterized by upregulated lipid metabolism promoting oxidative stress, suppressed glucose metabolism reducing energy availability, and restricted amino acid metabolism impairing the synthesis of effector molecules, which collectively contribute to NK cell dysfunction, leading to diminished proliferative capacity, impaired effector function, and reduced survival. In the following part, we will not only elaborate the alternation of NK cells in lipid metabolism, glucose metabolism, amino acid metabolism, but also discuss the causes for metabolic reprogramming of NK cells (such as hypoxia, tumor metabolites in TME, and the subsequent impact on their life activities and functions).

### 4.1 Glucose Metabolism

**FIGURE 2:**
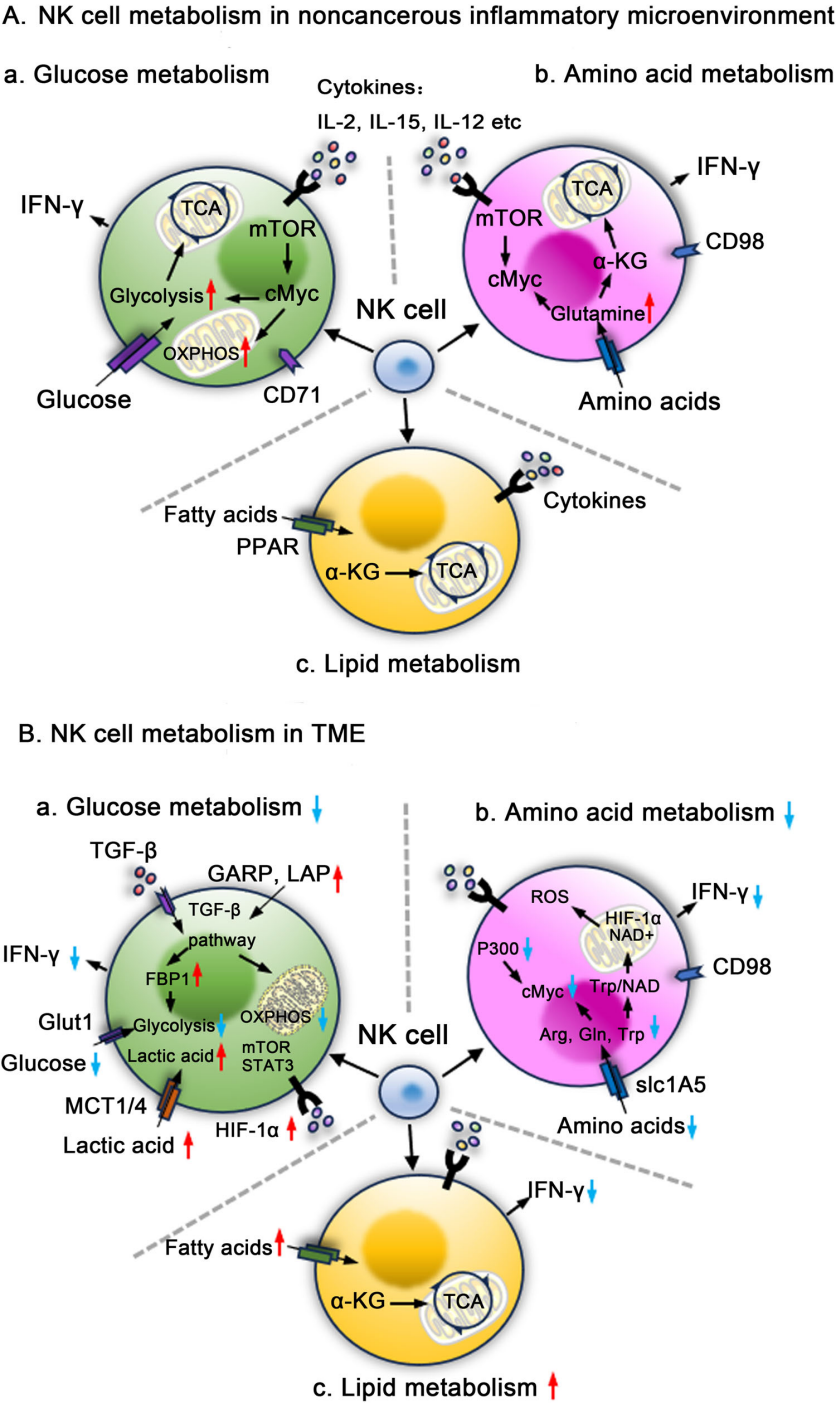
TME shapes NK cell metabolisms. As shown in panel (A), in the resting state, NK cells primarily rely on low‐level glycolysis and mitochondrial OXPHOS to sustain their basic physiological functions. Panel a: Upon stimulation with cytokine IL‐2 or the combination of IL‐12 and IL‐15, the levels of OXPHOS and glycolysis in NK cells are markedly increased, facilitating enhanced glucose uptake and supporting their normal function. Stimulation by IL‐2 activates the mTORC1 signaling pathway in NK cells, which promotes glycolysis and OXPHOS through cMyc and ultimately contributes to the regulation of IFN‐γ production. Panel b: Simultaneously, the expression of amino acid transporters SLC1A5 and CD98 on NK cell surfaces is significantly upregulated, indicating enhanced amino acid uptake to meet the demands of increased amino acid metabolism following activation. Additionally, the stability of cMyc translation requires amino acids, highlighting their role in this process. Panel c: To ensure an adequate energy supply, lipid metabolism plays a crucial role in the growth, differentiation, maturation, and function of NK cells. Lipid levels in NK cells are primarily regulated by the PPAR. Alpha‐ketoglutaric acid (αKG), an intermediate of the TCA cycle, is generated from the catabolism of fatty acids and glutamine, thereby supplying energy to NK cells. Panel (B) demonstrates that in the TME, three types of NK cell metabolism undergo alterations. Panel a: The glucose metabolism of NK cells, encompassing glycolysis and OXPHOS, is significantly reduced. On one hand, the superior metabolic capacity of tumor cells restricts the availability of glucose for NK cells within the tumor microenvironment. Conversely, the accumulation of lactate, along with the activation of signaling pathways such as TGFβ and STAT3, impairs the mitochondrial function of NK cells. Panel b: Similarly, amino acid metabolism in NK cells is constrained within the TME. As tumor cells preferentially utilize arginine, tryptophan, and glutamine, the metabolic levels of these amino acids in NK cells significantly decrease, thereby impairing their proliferation, cytotoxicity, and IFN‐γ production. Reduced tryptophan metabolism increases the susceptibility of NK cells to ROS‐induced damage. A reduction in glutamine levels within NK cells leads to a corresponding decrease in cMyc levels. Additionally, the accumulation of lipids results in reduced expression of acetylase P300, thereby hindering the acetylation of cMyc and ultimately impairing NK cell proliferation and antitumor function. Panel c: The buildup of ROS leads to oxidative stress, causing cellular damage. NK cells enhance lipid metabolism by increasing the absorption and storage of lipids within the lipid‐rich tumor microenvironment to counteract oxidative stress. However, this increase in lipid metabolism correlates with a decrease in IFN‐γ secretion.

Glucose metabolism serves not only as the primary energy production pathway for NK cells but also significantly influence their functional capacity. In resting state, NK cells primarily rely on glycolysis and intra‐mitochondrial OXPHOS at low levels to sustain their basic physiological functions [59]. However, upon external stimulation by cytokines such as IL‐2 or the combination of IL‐12 and IL‐15, NK cells in peripheral blood significantly upregulate their levels of OXPHOS and glycolysis to enhance glucose uptake and maintain normal cellular functions (Figure 2A‐a) [13]. Poznanski et al. [60] isolated NK cells from tumor samples of patients with ovarian cancer or lung cancer and compared them with peripheral blood NK cells. They found that the glucose metabolism ability of NK cells in both tumor and peripheral blood decrease with different degrees, while the antitumor function of NK cells is also correspondingly impaired [60]. Since the metabolic capacity of tumors are more competitive than NK cells, the glucose obtained by NK cells from TME is limited. Besides, multiple stressors in TME, such as tumor metabolic byproducts and dysregulated signaling pathways, disrupt NK cell glucose metabolism, ultimately impairing their immune function, mechanisms were as follows.

#### 4.1.1 Inhibition of Glucose Metabolism Mediated by Dysregulated Signaling Pathways

Several studies show that the glycolysis of NK cells is impaired due to the regulation of TGF‐β accumulated in the TME. TGF‐β induces the aberrant upregulation of fructose‐1, 6‐bisphosphatase 1 (FBP1), a rate‐limiting enzyme involved in gluconeogenesis, leading to the depletion of the glycolytic intermediate FBP. This process suppresses the activity of hexokinase 2 (HK2) and 6‐phosphofructo‐2‐kinase/FBP3, thereby impairing glycolysis of NK cell. In lung and ovarian cancer, TGF‐β‐mediated metabolic reprogramming is discovered and significantly reduces NK cell glycolytic capacity, accompanied by decreased IFN‐γ secretion and diminished release of cytotoxic granules [61, 62].

Moreover, TGF‐β not only directly suppresses NK cell glucose metabolism but also enhances this inhibitory effect by downregulating other pathways, such as the mTOR pathway [63]. When tracing back to the upstream, the upregulated expression of glycoprotein‐A repetitions predominant (GARP) and latency‐associated peptide (LAP) from TME forms complexes to activate the TGF‐β–Smad3 signaling pathway, further inducing mitochondrial fission to intensify the glucose metabolic suppression of NK cells [62].

#### 4.1.2 Inhibition of Glucose Metabolism Mediated by Tumor‐Derived Byproducts Depleting Energy Sources in the TME

Hypoxia and lactate accumulation drive mitochondrial fission in NK cells by inducing phosphorylation of Drp1 at Ser616, leading to disruption of the mitochondrial network, which characterized by reduced mitochondrial mass and impaired fusion capacity of activated mitochondria, ultimately compromising OXPHOS efficiency and diminishing NK cell‐mediated antitumor immune activity (see Figure 2B‐a for details) [60]. Moreover, vitamin B6 deficiency induced by the heightened metabolism of tumors impairs NK cells’ ability to degrade glycogen, rendering NK cells unable to rapidly acquire energy through glycolysis during the acute response [64].

#### 4.1.3 Inhibition of Glucose Metabolism by Other Metabolic Abnormalities

Concurrently, lipid peroxidation byproducts (e.g., 4‐hydroxy‐2‐nonenal [4‐HNE]) generated from dysregulated lipid metabolism in NK cells not only covalently modify mitochondrial complex I (NDUFS2 Cys39) and inhibit GLUT1 membrane trafficking but also inhibits mTORC1 signaling through the activation of the JNK pathway, while mTORC1 inactivation further downregulates the expression of glycolytic enzymes [65]. This establishes a self‐reinforcing loop—lipid peroxidation begets glucose metabolic suppression—which depletes ATP reserves and perpetuates NK cell dysfunction [60].

In summary, TGF‐β‐induced mitochondrial fragmentation, together with hypoxia‐ and lactate‐driven Drp1 activation, collectively leads to the collapse of OXPHOS. As a result, NK cells are forced to transiently rely on inefficient glycolysis to sustain survival and compensatory autophagy to maintain immune function in the short term [62, 66, 67, 68]. However, long‐term glucose metabolism inhibition not only progressive exhaust NK cell‐mediated antitumor activity, specifically manifested as reduced IFN‐γ secretion and impaired cytotoxic granule release, but also induces H3K27me3‐mediated epigenetic silencing, leading to the permanent shutdown of proliferation‐related genes, which results in a decline in proliferative capacity [64, 69], thereby resulting tumor progression or the failure of antitumor therapy.

### 4.2 Lipid Metabolism

Lipid metabolism plays a crucial role in the growth, differentiation, maturation, and function of NK cells. During the differentiation of mouse CD27^+^CD11b^−^ NK cells to CD27^−^CD11b^+^ NK cells, the expression levels of lipid synthesis‐related enzymes increase to ensure an adequate energy supply [70]. When stimulated by IL‐15, NK cells in peripheral blood undergo cholesterol metabolism reprogramming, leading to a significant increase in cholesterol levels on their cell membranes [71]. The myocyte enhancer factor 2C (MEF2C) transcription factor coordinates IL‐2‐ and IL‐15‐induced activation of the sterol regulatory element‐binding protein (SREBP) pathway, promoting cholesterol and FA synthesis. This regulation preserves NK cell membrane fluidity and signal transduction. MEF2C deficiency disrupts lipid metabolism and markedly impairs NK cell cytotoxicity and cytokine secretion [72]. However, excessive lipid accumulation in NK cells of obese mice and humans disrupts cell metabolism and impairs normal function (Figure 2A‐c) [73]. It has been reported that one of the hallmarks of tumors in a high metabolic state is the elevated production of reactive oxygen species (ROS). The accumulation of ROS in the TME induces oxidative stress and results in varying degrees of cellular damage. To cope with this, tumor cells have evolved multiple strategies to counteract oxidative stress. Besides upregulating antioxidant enzymes through the Nrf2 signaling pathway, tumor cells can also reprogram lipid metabolism to create lipid‐rich microenvironment that confers protection against ROS‐induced damage [74, 75, 76].

Specifically, tumor cells enhance lipid metabolism by increasing the uptake and storage of peripheral lipids through the overexpression of FA transporter molecules (CD36, MSR1, and CD68), upregulating FAs synthesis and promoting FAO to support membrane biosynthesis and signal transduction [77, 78, 79]. Absorbed FAs are stored in lipid droplets (LDs), which not only directly neutralize ROS within tumor cells but also interact with various antioxidant enzymes upon mitochondrial transport, generating NADPH to further suppress ROS production.

However, unlike B and T cells, NK cells lack a comprehensive antioxidant enzyme system (e.g., PRDX1 and GPX1) to neutralize ROS, rendering them highly susceptible to oxidative stress [80]. Prolonged exposure to ROS profoundly impairs their viability and cytotoxic function, ultimately contributing to immune evasion and tumor progression, which could explain the failure of antitumor therapies targeting NK cell‐mediated immunity [81, 82]. In such a harsh TME, recent studies have revealed that NK cells are forced to increase lipid metabolism by enhancing absorption and storage of lipids in LDs as a temporary protective mechanism against oxidative damage [83, 84]. However, with the increase of lipid metabolism, the cytotoxic function and viability ability of NK cell are inevitably impaired [83, 85]. Within NK cells, the increased lipid peroxidation driven by elevated lipid metabolism not only directly impairs NK cell function but also generates metabolic byproducts such as 4‐HNE to inhibit GLUT1 membrane localization, further exacerbating glucose uptake deficiencies and perpetuating a vicious cycle of metabolic compensation [60]. In addition, mutual influence also exists among various metabolic pathways of NK cells. For instance, lipid levels in NK cells are primarily regulated by the peroxisome proliferator‐activated receptor (PPAR), and PPARα/δ inhibitors can restore normal levels of mTOR‐mediated glycolysis in NK cells hindered by lipid accumulation [73]. Furthermore, excessive intracellular lipid accumulation in NK cells leads to hypoacetylation of histone proteins, primarily affecting chromatin accessibility and transcription of effector genes. This epigenetic remodeling contributes to functional exhaustion, further limiting NK cell‐mediated antitumor immunity [85]. Moreover, the accumulated ferrous iron (Fe^2+^) intercepted from the TME will further catalyze the peroxidation of polyunsaturated fatty acids (PUFAs) through the Fenton reaction in NK cells, triggering ACSL4‐dependent ferroptosis (see Figure 2B‐c for details). Ferroptosis not only disrupts NK cell membrane integrity but also suppresses immune synapse formation via the release of oxidized phospholipids, ultimately leading to the loss of cytotoxic function [86, 87].

Together, the elevated accumulation of ROS in tumor immune microenvironment could cause damage to cells. Since lack of antioxidant enzyme system, NK cells utilize resources in the microenvironment to change metabolic patterns to prevent themselves from ROS damage. The tumor's high lipid metabolism status results in a lipid‐rich microenvironment and NK cells can adapt and survive briefly by increasing lipid metabolism by intaking and storing lipids. However, such a pattern obviously unable to bring profound benefits and form a vicious cycle of metabolic compensation. The progressive accumulation of lipids drives NK cell dysfunction, thereby compromising their antitumor immunity, which can also explain the failure of antitumor therapy in some patients due to the dysfunction of NK cells.

### 4.3 Amino Acid Metabolism

Amino acid metabolism is also tightly associated with functionality of NK cells. Jensen et al. [88] found that the activated mTORC1 signaling pathway in NK cells can regulate IFN‐γ production through myelocytomatosis oncogene (c‐Myc) under IL‐2 stimulation. Simultaneously, the expression of amino acid transporters SLC1A5 and CD98 on the surface of NK cells in peripheral blood increased significantly, suggesting that NK cells enhanced amino acid uptake to meet the high metabolic demands following activation [88]. Notably, amino acids are essential for the stabilization of cMyc translation levels, and a deficiency in Gln leads to impaired NK cell growth and function [89]. If l‐kynurenine (l‐Kyn) from the external environment enters the cell via the SLC7A5 transporter, it inhibits NK cell proliferation, cytokine secretion, and cytotoxicity (Figure 2A‐b) [90]. Hence, amino acid metabolism is vital for NK cells to sustain growth, cellular activity, and functional performance.

Amino acid deprivation within TME represents another key mechanism underlying NK cell functional exhaustion. Tumor cells competitively uptake essential amino acids such as arginine (Arg), tryptophan (Trp), and Gln, thereby forcing NK cells into a metabolically quiescent state. This metabolic deprivation not only restricts the biosynthetic capacity of NK cells but also suppresses their immune function through a dual mechanism involving epigenetic remodeling and dysregulation of key signaling pathways [91, 92, 93]. The following sections systematically elucidate these mechanisms from the perspective of aberrant metabolism of critical amino acids.

#### 4.3.1 Arg Metabolism

Arg is a critical substrate for NK cell synthesis of nitric oxide (NO) and polyamines, with its metabolic homeostasis tightly regulated by the balance of arginase‐1 (Arg1) activity within the microenvironment. Angka et al. [94] found that the postoperative inflammatory environment in colorectal cancer induces a significant increase in myeloid‐derived suppressor cells (MDSCs), which heavily consume Arg in the TME by synthesizing and accumulating Arg1, leading to impaired proliferation, cytotoxicity, and IFN‐γ production of NK cells, ultimately promoting postoperative tumor metastasis. However, postoperative administration of an Arg‐enriched diet could restore the activity and functionality of NK cells, thereby preventing the metastasis. Steggerda et al. [95] restored the proliferative capacity and antitumor activity of NK cells by blocking the expression of Arg1 from myeloid cells in TME. Additionally, Lamas et al. [96] and Westhaver et al. [97] also confirmed in vitro experiments that the proliferative and immunological functions of NK cells depend on the supply of Arg. Arg deprivation suppresses NK cell function through the following mechanisms: first, Arg deficiency restricts NO production, thereby weakening the direct cytotoxicity of NK cells against tumor cells [96]; second, as an essential substrate for mTORC1 activation, Arg depletion leads to mTORC1 inactivation, which subsequently inhibits glycolysis and suppresses the synthesis of cytotoxic granules, including perforin and granzyme B [98]; third, Arg regulates the activity of the histone methyltransferase PRMT5, which maintains the H3R8me2 modification at the −54 kb enhancer region of the IFN‐γ gene promoter. Its depletion disrupts this epigenetic regulation, leading to transcriptional silencing and reduced IFN‐γ secretion [99, 100].

#### 4.3.2 Trp Metabolism

Trp is essential for the survival and immunological function of NK cells [101] and metabolized through the Kyn pathway to NAD+, which serves as a key metabolic axis for NK cells to resist oxidative stress from TME (see Figure 2B‐b for details) [102]. However, tumors exploit high expression of indoleamine 2, 3‐dioxygenase (IDO)1/Trp‐2, 3‐dioxygenase (TDO)2 to convert Trp into Kyn, leading to dual damage. On the one hand, Trp deprivation limits NAD+ synthesis [103, 104], inhibiting SIRT1‐dependent mitochondrial deacetylation repair functions, exacerbating ROS‐induced mitochondrial damage [105]; on the other hand, the accumulated Kyn activates the aryl hydrocarbon receptor (AhR), inducing programmed cell death protein 1 (PD‐1) transcription and suppressing NKG2D expression, thus forming a “metabolic‐checkpoint” synergistic inhibition [106, 107]. Although tumor cells can utilize phenylalanine (Phe) as a substitute for Trp in protein synthesis, NK cells are unable to initiate compensation due to the lack of Phe hydroxylase, ultimately leading to metabolic stagnation.

#### 4.3.3 Gln Metabolism

Gln serves as a crucial metabolite for maintaining epigenetic activity in NK cells, primarily by stabilizing the c‐Myc protein. This process is facilitated through multiple mechanisms, including the provision of metabolic intermediates, activation of signaling pathways such as mTORC1, regulation of protein synthesis, and promotion of c‐Myc transcription [90, 101]. Furthermore, c‐Myc plays a pivotal role in maintaining chromatin accessibility at the promoter regions of key immune‐related genes, such as IFN‐γ, PFN, and GZMB, by recruiting the histone acetyltransferase P300, thereby supporting NK cell immune functions [85].

In TME, tumor cells preferentially uptake Gln from the microenvironment for energy metabolism through the overexpression of SLC1A5 and SLC7A5 Gln transporters, thereby forcing NK cells into a metabolic crisis [90, 108, 109]. The resulting Gln depletion accelerates c‐Myc degradation via the ubiquitin–proteasome pathway, impairing both NK cell proliferation and antitumor functions [90, 108]. Moreover, since c‐Myc is a key upstream regulator of aerobic glycolysis genes such as GLUT1, HK2, ENO1, PKM2, and LDHA [110], the reduction in c‐Myc levels within NK cells due to Gln deprivation further impairs their glycolytic metabolism, exacerbating the loss of NK cell proliferation and immunological functions.

Interestingly, the functionality of the c‐Myc prote‐in is regulated not only by a single metabolic pathway. The enhanced lipid metabolism in NK cells can also leads to lipid accumulation‐induced chromatin remodeling, further reducing the expression of the histone acetyltransferase P300 [85].Consequently, the decreased expression of P300 impairs the functionality of c‐Myc, leading to its degradation and subsequently inhibiting NK cells from synthesizing perforin and granzyme B (see Figure 2B‐b for details).

#### 4.3.4 Other Amino Acids Metabolism

Furthermore, Zheng et al. [111] found that dysregulation of serine metabolism in NK cells can impair the biosynthesis of cell surface phospholipids and inhibit the formation of membrane protrusions, which prevents NK cells from forming immunological synapses, thereby compromising their ability to eliminate tumors.

### 4.4 Metabolic Crosstalk: Immunoregulatory Roles of TCA Cycle Intermediates

Beyond glucose metabolism, amino acid and lipid metabolism function synergistically to sustain NK cell immunological activity. Intermediates of the TCA cycle, such as α‐ketoglutarate (α‐KG), can be produced through the catabolism of FAs and Gln respectively, suppling energy to NK cells [14]. The expression of carnitine palmitoyl transferase I (CPT1A), a key enzyme in FAO, is upregulated in response to viral infection, enhancing the metabolic activity of NK cells [112]. These findings indicate that products of lipid and amino acid metabolism, as well as the relevant transporter proteins, may help maintain stable levels of OXPHOS and glycolysis, which in turn supported adequate energy for immune function of NK cells. The metabolic homeostasis of NK cells is therefore dynamic and subject to fluctuation in response to these factors. Additionally, there is mutual influence among the various metabolic pathways of NK cells, contributing to a highly regulated and context‐dependent metabolic response. The functional state of NK cells fundamentally reflects their metabolic plasticity, while the interplay between synergistic and antagonistic metabolic pathways dictates the strength and persistence of the immune response.

## 5 Regulation of NK Cell Function by Epigenetic Mechanisms

As research into NK cell immune responses continues to advance, the pivotal role of epigenetic regulation has gained wide recognition. The following sections will elaborate on the mechanisms by which epigenetic factors regulate NK cell effector functions, focusing on three key aspects: DNA acetylation and methylation, histone modification, and the influence of noncoding RNAs.

### 5.1 DNA Acetylation, Methylation, and Histone Modifications

Although early studies have shown that the rapid production of IFN‐γ by NK cells upon stimulation is independent of their proliferation, and that proliferation‐dependent chromatin remodeling does not significantly affect cytokine and chemokine production by NK cells [113], the regulatory role of DNA acetylation and methylation in NK cell effector functions—such as acetylation and methylation of the IFNG locus and methylation of KIR genes—has been clearly demonstrated and warrants further investigation.

Even in the absence of external stimulation, both murine and human NK cells possess an open and accessible chromatin structure at the IFNG locus, which remains transcriptionally active and continuously produces low levels of IFN‐γ transcripts [113, 114]. These basal transcripts are thought to prime NK cells for a rapid IFN‐γ response upon stimulation. Trimethylation of histone H3 lysine 4 (H3K4me3) at the IFNG locus facilitates IFN‐γ expression. Increased expression of the long noncoding RNA Tmevpg1 promotes broader H3K4me3 enrichment at the IFNG locus, thereby enhancing gene activation and augmenting IFN‐γ production [115]. Upon stimulation with IL‐12, acetylation of histone H4 within the first intron of IFNG is elevated, facilitating transcription factor recruitment and promoting IFN‐γ transcription and NK cell‐mediated immune responses [116, 117].

As discussed in Section 3, due to the high polymorphism of class I HLA molecules, which serve as ligands for KIRs, the KIR gene locus is highly diverse among individuals, and the number of NK cells expressing distinct KIR genes also varies within the same individual. For example, peripheral blood NK cells expressing KIR3DL1 show low DNA methylation in the promoter region but high methylation in the first exon and intron, suggesting a region‐specific methylation pattern linked to transcriptional regulation. Although the exact mechanisms remain unclear, modulation of KIR methylation through targeting DNA methyltransferases may offer a new strategy to influence KIR expression and NK cell diversity [118].

In addition to the impact of DNA methylation on inhibitory receptors, the methylation status of activating receptors and their ligands—such as NKG2D and its ligands MICA and MICB—also plays a regulatory role in NK cell‐mediated immune responses [119]. In NK cells isolated from patients with HCC, the promoter region of NKG2D exhibits significantly higher levels of methylation compared with healthy controls. Some researchers have even proposed NKG2D promoter methylation as a potential biomarker for HCC [120]. In patients with AML, hypermethylation of the NKG2DL promoter has been shown to impair its transcription and reduce binding affinity to NKG2D, ultimately compromising NK cell recognition and immune function. These findings support the therapeutic potential of DNA demethylation strategies to enhance NK cell antitumor immunity [119, 121].

### 5.2 Noncoding RNAs

The regulatory roles of noncoding RNAs in NK cell function have been increasingly elucidated and refined with advancing research. Both miR‐29 and miR‐155 modulate IFN‐γ expression and thereby affect the effector function of NK cells. MiR‐29 suppresses IFN‐γ translation, while miR‐155 enhances IFN‐γ production [114]. Upon stimulation with IL‐12 and IL‐18, both human and murine NK cells activate NF‐κB signaling, which promotes IFN‐γ transcription, and concurrently phosphorylated STAT4 binds to the upstream promoter region of the miR‐155 gene to induce its expression [122]. Overexpressed miR‐155 binds to the 3′ untranslated region of SHIP1, a negative regulator of IFN‐γ, thereby suppressing SHIP1 translation, activating the PI3K pathway, and further promoting IFN‐γ production [123]. Moreover, miR‐155 can also bind to transcripts of proapoptotic molecule Noxa and suppressor of cytokine signaling 1, thereby supporting NK cell immune responses through multiple regulatory axes [122]. As research progresses, increasing attention has also been paid to other noncoding RNAs such as miR‐223 and miR‐29, which are involved in the regulation of NK cell‐mediated cytotoxicity and cytokine/chemokine secretion against tumors [124, 125].

In addition to their critical roles in regulating NK cell immune function, noncoding RNAs are also indispensable for NK cell maturation and development. During NK cell maturation, the transcript levels of miR‐150 and miR‐181 progressively increase, reflecting their essential involvement in this process. Deletion of either miR‐150 or miR‐181 results in a decreased proportion of mature NK cell subsets. miR‐150 promotes NK cell maturation by targeting the negative regulator of cell development, c‐Myc. In its absence, NK cells are arrested in an immature state [126]. Similarly, miR‐181 facilitates early NK cell development by targeting Nemo‐like kinase, thereby activating the Notch signaling pathway [127].

## 6 The Impact of the TME on NK Cell Function

TME refers to the peripheral environment in which tumors exist, encompassing surrounding blood vessels, various immune cells, fibroblasts, inflammatory cells, various effector molecules and signaling pathways, metabolic byproducts, extracellular matrix, and so forth [128]. TME is a dynamic ecosystem that forces NK cells into a metabolic compensation state through multiple mechanisms, including hypoxia, metabolic byproducts, and cellular interactions, which also serves as the driving force behind the metabolic reprogramming of NK cells, as previously discussed, involving lipid peroxidation, glycolysis inhibition, and amino acid depletion [129, 130, 131]. Subsequent sections will systematically dissect the impact of various factors within the TME on NK cell metabolism, along with the corresponding functional exhaustion.

### 6.1 Physical Factors: Hypoxia, Acidosis, Nutrient Deprivation (Focus on HIF‐1α and Beyond)

The tumor's increased oxygen consumption and immature vascular oxygen delivery system contribute to a sustained hypoxic TME, which stabilizes HIF‐1α by inhibiting prolyl hydroxylase (PHD)‐mediated degradation, leading to its accumulation. [132]. As a core feature of the high metabolic state of tumors, hypoxia is not only a direct consequence of metabolic abnormalities in the TME but also an initiating signal for NK cell metabolic reprogramming. In normoxic conditions, HIF‐1α does not exert regulation over the glycolytic and OXPHOS pathways in NK cells [90]. However, HIF‐1α’s role in NK cells within remains controversial [133]. The reported mechanisms underlying the effects of exposure to HIF‐1α on NK cell metabolism and function are as follows.

#### 6.1.1 HIF‐1α Suppresses NK Cell Metabolism and Impairs Their Immune Function

Some researchers have revealed that the elevation of HIF‐1α in the TME leads to a reduction in NK cell metabolism, consequently impairing their immune function. Ni et al. [134] discovered that the deletion of the HIF‐1α gene in mouse NK cells and subsequent culture under hypoxic conditions result in enhanced glycolysis, increased oxygen consumption, and secretion of IFN‐γ. In mouse tumor models under hypoxic conditions, the growth of tumors in HIF‐1α knockout mice is suppressed. Correspondingly, single‐cell sequencing results show high expression of genes representing NK cell immune function, such as IFN‐γ and granzyme B with deletion of the HIF‐1α. These findings indicate that HIF‐1α inhibits the energy metabolism and immune function of NK cells under hypoxic conditions, which aligns with the downregulation of signaling molecules responsible for activating NK cell immune function (such as NKp30, NKp44, NKp46, NKG2D receptor and CD16) in the TME revealed by other research [135, 136].

#### 6.1.2 HIF‐1α Enhances NK Cell Metabolism and Impairs Their Immune Function

However, recent research presents contrasting perspectives. The study conducted by Pelletier et al. [102] posits a beneficial role for HIF‐1α in regulating both the metabolic and immune functions of NK cells. They observed that HIF‐1α orchestrates Trp/NAD+ metabolism in resting NK cells, shielding them from ROS damage. Moreover, under hypoxic conditions, HIF‐1α not only directs the metabolic shift of activated NK cells from OXPHOS to glycolysis but also bolsters their immune response by stimulating and augmenting glycolysis [102]. Similarly, Krzywinska et al. [137] found that the absence of HIF‐1α in hypoxic settings hinders NK cells’ ability to efficiently engage in glycolysis for energy acquisition, thereby impairing their immune function. Cluff et al.’s [138] study similarly argued that HIF‐1α could activate the cytotoxicity and IFN‐γ production of NK cells. They found that NK cells isolated from healthy individuals fail to produce HIF‐1α upon IL‐2 activation, no matter hypoxic and normoxic conditions, and the cytotoxicity of these NK cells were impaired [138]. However, after being cultured in vitro, NK cells produce significant amounts of HIF‐1α upon IL‐2 stimulation under hypoxic conditions, and concomitantly, their cytotoxicity markedly increase. Based on these findings, they postulated that HIF‐1α could not maintain stability to activate NK cell function since it could be hydroxylated by PHDs and prone to proteasomal degradation in a normoxic environment. Even under hypoxic conditions, the redistribution of intracellular oxygen due to the inhibition of mitochondrial aerobic processes further strengthens the degradation of HIF‐1α by PHDs [139]. In contrast, NK cells expanded in vitro and stimulated with IL‐2 could continuously activate the synthesis of HIF‐1α through the activation of the PI3K/mTOR pathway, thereby enhancing NK cell immune function.

#### 6.1.3 The Role of HIF‐1α in NK Cells May be Shaped by the Dynamic Conditions of the TME

Based on these studies and findings, we assume that the controversy involving the role of HIF‐1α in regulating NK cell metabolism and immune function may stem from its interaction with other TME factors such as nutrient scarcity and immunosuppressive metabolites (e.g., ADO, lactate). First, HIF‐1α’s ability to enhance glycolysis is nullified under glucose/Gln scarcity. For example, HIF‐1α‐driven glycolysis requires SLC2A1 (GLUT1)‐mediated glucose uptake, which is suppressed in nutrient‐poor TME. This explains why hypoxia‐activated HIF‐1α improves NK cell metabolic fitness and immune function in nutrient‐replete in vitro conditions. Second, from a chronic TME adaptation perspective, hypoxia‐induced HIF‐1α exhibits dual immunosuppressive effects: on one hand, it upregulates LDHA to increase lactate production, which suppresses NK cell cytotoxicity via H3K18 lactylation (H3K18la)‐mediated IFNG silencing, forming a feedforward loop; on the other hand, HIF‐1α transactivates CD73 to promote ADO generation, and ADO–A2AR signaling further inhibits AMPK/mTORC1 activity, exacerbating metabolic paralysis [140, 141]. Therefore, we propose a context‐dependent model: in acute hypoxia (short‐term oxygen fluctuations) or in vitro nutrient sufficiency, HIF‐1α supports NK cell survival and activation, whereas in chronic hypoxia (prolonged oxygen deprivation), combined with resource exhaustion and activation of tumor‐promoting signaling pathways, HIF‐1α drives NK cell metabolic dysfunction.

Additionally, we suggest that future studies on the role of HIF‐1α in regulating NK cell metabolism and immune function could conduct exploratory experiments from short‐term to long‐term perspectives to obtain reliable validation. Additionally, we suggest that most of the research regarding the positive regulation of NK cells by HIF‐1α has predominantly been conducted in vitro, thereby overlooking the factor of nutrient deprivation within the TME during experimental procedures. Even if HIF‐1α exhibits stimulatory effects on the immune function of NK cells, the scarcity of energy and nutrients could hinder the successful activation of NK cells by HIF‐1α. Hence, future investigations require comprehensive validation of the role of HIF‐1α in NK cells through systematic in vivo and in vitro studies.

### 6.2 Chemical Factors: Immunosuppressive Metabolites

#### 6.2.1 Lactate

Another important feature of the TME is the substantial accumulation of lactate resulting from tumor aerobic glycolysis, known as the Warburg effect [142]. In the TME, lactate concentrations can reach levels as high as 10–30 mM, which can be 5–10 times higher than those in normal physiological conditions [143]. Lactate inhibits NK cell activity and immune function through a dual mechanism: on the one hand, the accumulation of lactic acid diminishes the cytotoxic and secretory functions by downregulation expression of activated receptors such as NKp46, CD107a and NKG2D of NK cells, thereby blocking tumor recognition signals [144, 145, 146]. On the other hand, lactate accumulation creates a low pH environment surrounding NK cell, which prevents NK cells from metabolically extruding intracellular lactate along the lactate concentration gradient (by membrane channel protein MCT1–4), leading to intracellular lactate accumulation. Consequently, intracellular lactate accumulation not only inhibits HK2 activity and glycolytic flux, leading to H3K18la modification and silencing of IFNG/GZMB expression, thereby impairing the immune secretory function of NK cells [147, 148], but also induces an NADH/NAD+ imbalance, which inhibits complex I activity (oxidation of NDUFS2 Cys39), resulting in mitochondrial stress and subsequent cell death [149].

Additionally, the accumulation of lactate, in conjunction with hypoxia, activates HIF‐1α, which further upregulates CD73 to promote ADO generation. This forms a “hypoxia–lactate–ADO” immune‐suppressive positive feedback loop, which further reshapes NK cell metabolism and accelerates functional exhaustion [148].

#### 6.2.2 ADO

In normal extracellular environment, exogenous ADO can be rapidly taken up into the cells via nucleoside transporters and swiftly metabolized by ADO deaminase, which leads to rapid phosphorylation into AMP or degradation into inosine, thereby maintaining low concentrations. However, hypoxia, acidity, and nutrient deprivation within the TME lead to extensive cell death, resulting in the release of ATP into the extracellular space. Tumor cells overexpress ectonucleotidases CD39, which hydrolyzes ATP to AMP, and CD73, which converts AMP to ADO, thereby establishing an ADO‐generating cascade that elevates ADO concentrations in the TME up to 100 times higher than those in normal tissues. Furthermore, hypoxia‐inducible factor 1‐alpha (HIF‐1α) enhances the expression of CD39 and CD73, further exacerbating ADO accumulation [150, 151, 152, 153]. The mechanisms currently elucidated that ADO, by binding to the A2A receptor, not only directly inhibits the proliferation and maturation of NK cells but also downregulates the expression of activating receptors NKG2D and NKp30 to restrict NK cell activation [154, 155]. Additionally, ADO can recruit and activate immune‐suppressive cells such as T‐reg cells and MDSCs to indirectly suppress the immune function of NK cells [156]. In addition, ADO can further exacerbate the suppression of NK cell immune function by reprogramming their metabolism. Specifically, ADO can attenuate glycolysis and OXPHOS rates in NK cells by inhibiting the STAT5 and mTOR pathways activated by IL‐12 and IL‐15, thereby impairing NK cell cytotoxicity [157].

#### 6.2.3 Other Metabolites

The heightened tumor metabolism of Trp through the IDO or TDO pathways result in the accumulation of the metabolic byproduct l‐Kyn in the TME. Elevated concentrations of l‐Kyn are then transported into NK cells via amino acid transporters (SLC1A5, SLC3A2/SLC7A5). Intracellular excessive l‐Kyn can induce the expression of PD‐1 and TIM‐3 by activating the AhR, while simultaneously downregulating NK cell activation receptors (such as NKG2D and NKp46), thereby establishing a “metabolic–checkpoint” synergistic inhibition that impairs NK cell immune function [158, 159, 160]. Furthermore, the activation of AhR exacerbates NAD⁺ depletion and subsequently inhibits SIRT1‐dependent mitochondrial autophagy of NK cells, thereby mediating its ROS‐induced apoptosis [101, 160, 161].

### 6.3 Cellular Crosstalk: Interactions with Tumor Cells and Other Immune Cells

**FIGURE 3:**
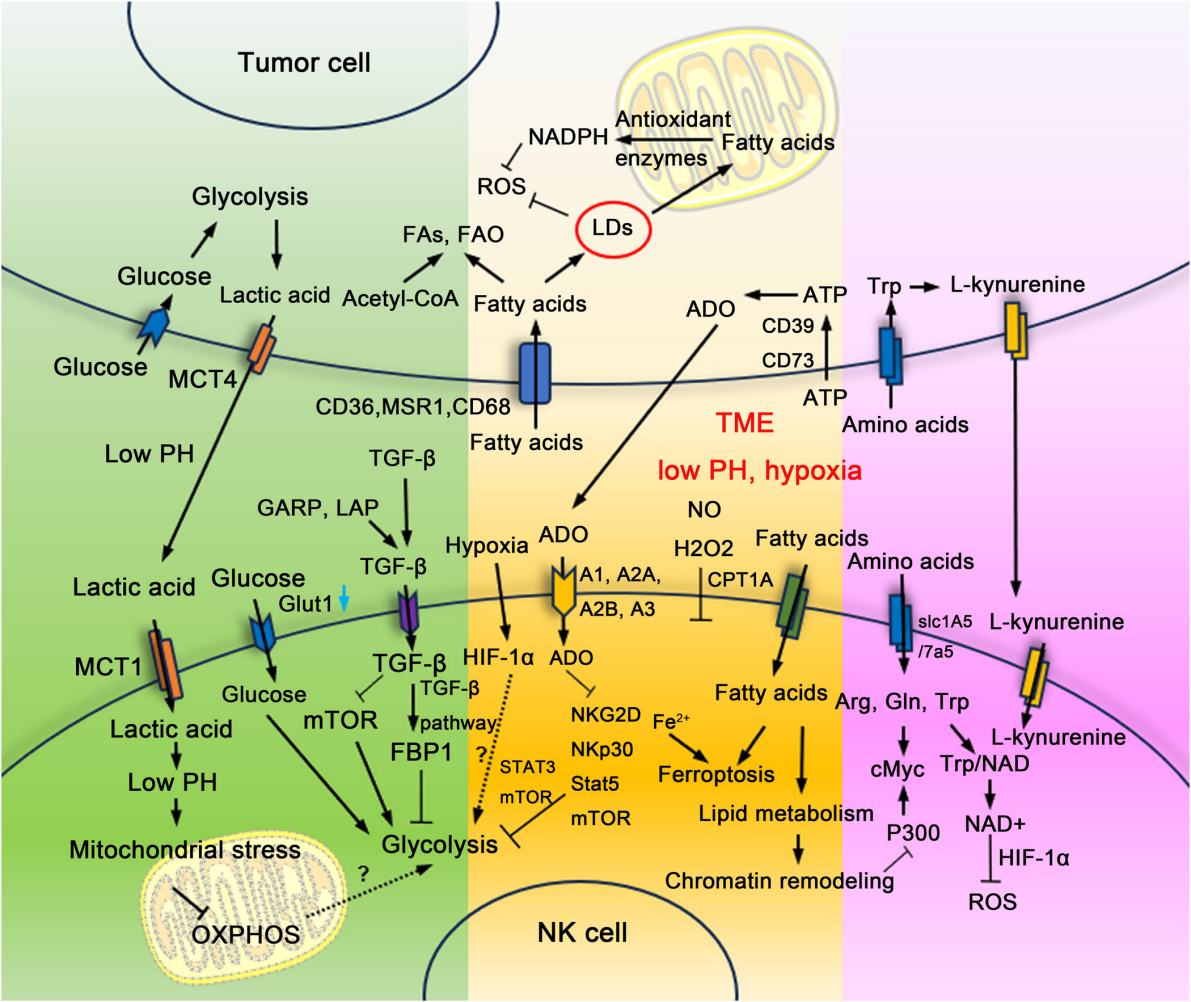
Signaling pathways involved in metabolism reprogramming of NK in TME. The green section of this figure illustrates byproducts of the elevated metabolism in tumor cells, including hypoxia, lactic acid accumulation, and the activation of signaling pathways like TGFβ and STAT3, can impair mitochondrial function in NK cells, leading to a significant reduction in their glucose metabolism. On one hand, the impaired glycolytic function of NK cells is attributed to the reduced expression of Glut1, the glucose transporter on the NK cell membrane, and the restricted availability of glucose in the TME. Conversely, the accumulation of TGF‐β in the TME induces an aberrant increase in FBP1, thereby inhibiting glycolysis. Meanwhile, the increased expression of GARP and LAP activates the TGFβ signaling pathway while inhibiting the mTOR pathway, thereby reducing NK cell metabolism. The substantial accumulation of lactic acid produced by tumor cell glycolysis creates a low pH environment around NK cells, leading to lactate buildup within these cells. This accumulation induces mitochondrial stress, disrupting the OXPHOS process and potentially leading to cell death. The yellow section of this figure illustrates that during tumor development and progression, tumor cells modulate lipid metabolism to meet the demands of new cell membrane synthesis and signal transduction. This regulation includes increased uptake and storage of peripheral lipids via overexpression of fatty acid transport molecules (CD36, MSR1, and CD68), enhanced FAs synthesis, and increased FAO utilizing acetyl‐CoA from other metabolic pathways. Conversely, fatty acids absorbed and stored in lipid droplets (LDs) can directly neutralize the ROS response within tumor cells and also interact with antioxidant enzymes in the mitochondria to generate NADPH, thereby inhibiting ROS production. To counteract the damaging effects of oxidative stress, NK cells similarly upregulate lipid metabolism by increasing lipid absorption and storage. Within the TME, NK cells enhance their metabolic flexibility by increasing fatty acid uptake and upregulating the expression of carnitine palmitoyl transferase I (CPT1A) to promote fatty acid oxidation. Accumulated Fe^2+^ in NK cells react with FAs through lipid peroxidation, which induces ferroptosis. Exposure to NO and H_2_O_2_ impairs the cytotoxicity of NK cells and inhibits their activity. Following cell death in the TME, significant amounts of ATP are released. Tumor cells overutilize this ATP via the exonuclides CD39 and CD73, promoting its conversion to ADO. Subsequently, ADO accumulated in the TME binds to G protein‐coupled adenosine receptors (A1, A2A, A2B, and A3) on NK cells. This binding directly inhibits NK cell proliferation and maturation, while also downregulating the expression of activating receptors NKG2D and NKp30, thereby limiting NK cell activation. In addition, ADO can also reduce the glycolysis and OXPHOS rate of NK cells by inhibiting the IL‐12 and IL‐15 activated STAT5 and mTOR pathways, thereby impelling the cytotoxicity of NK cells. Hypoxia within the TME induces the expression of the HIF family, particularly HIF‐1α, which subsequently activates STAT3 and mTORC1, thereby upregulating glycolytic pathways in response to hypoxic conditions. The impact of enhanced HIF‐1α expression on NK cell function within the TME remains controversial. The purple section of this figure illustrates that tumor growth necessitates the absorption of a significant number of amino acids for protein synthesis, leading to a tumor microenvironment marked by amino acid deficiency, which in turn constrains the normal amino acid metabolism of NK cells. The excessive consumption of arginine within the TME impairs NK cell proliferation, cytotoxicity, and IFN‐γ production. NK cells utilize nicotinamide adenine dinucleotide (NAD+) generated by the tryptophan/NAD metabolic pathway in conjunction with HIF‐1α to counteract damage caused by accumulated ROS in the TME. The high amino acid metabolism of tumor cells intensifies tryptophan consumption within the TME. Furthermore, the enhanced metabolism of tryptophan by tumor cells results in the accumulation of l‐kynurenine in the TME. The elevated concentration of l‐kynurenine is transported to NK cells through the SLC7A5 amino acid transporter, which further inhibits NK cell proliferation, cytotoxic activity, and cytokine secretion. Tumor cells and NK cells exhibit differing requirements and absorption capacities for glutamine in the TME, leading to the preferential consumption of glutamine by tumor cells. The equilibrium between cMyc synthesis and degradation in NK cells is dependent on the availability of glutamine. Reduced glutamine levels consequently decrease cMyc levels, impairing NK cell proliferation and antitumor function. The acetylation of cMyc initiates the transcription and expression of IFN‐γ, PFN, and GZMB genes, thus facilitating the immune function of NK cells. The acetylase P300, which acetylates cMyc, is influenced by lipid metabolism. Enhanced lipid metabolism in NK cells induces chromatin remodeling due to lipid accumulation, subsequently reducing the expression of histone acetyltransferase P300.

In addition to soluble metabolites, stromal cells within the TME further hijack NK cell metabolism through direct metabolite transfer or secretion of regulatory factors. For example, in breast cancer, lung‐resident mesenchymal cells deliver lipids to NK cells via exosomes rich in PUFAs, thereby triggering ACSL4‐dependent lipid peroxidation to inhibit NK cell cytotoxic function [162]. On the other hand, polarized M2 macrophages consume Arg via Arg1, mimicking the effects of Gln deprivation and promoting the activation of TGF‐β to inhibit the mTORC1 pathway, which disrupts NK cell mitochondrial respiration and, in concert with ADO, suppresses IFN‐γ secretion [163]. These cellular components, along with metabolic byproducts such as ADO and lactate, together constitute the “metabolic immune suppression network” that modulates NK cell function within TME. (Factors and signaling pathways involved in metabolism‐related interactions between NK cells and tumor cells in TME were described and summarized in Figure 3.)

## 7 Clinical Translation and Therapeutic Strategies Targeting NK Cell Antitumor Immunity

NK cell‐based therapy has emerged as a prominent focus in the field of antitumor therapy due to its broad cytotoxic range, HLA‐unrestricted killing ability, rapid response, cost effectiveness, and high safety profile (low risk of immune rejection). Recent years, multiple therapeutic strategies have been developed to harness and enhance NK cell‐mediated antitumor immunity, which can be broadly classified into six major categories: (1) adoptive NK cell therapy, (2) targeting the TME to restore or augment NK cell function, (3) metabolic modulation to enhance NK cell persistence and cytotoxicity, (4) immune checkpoint blockade tailored for NK cells, (5) cytokine‐based therapies and receptor agonists, and (6) NK cell engagers (NKCEs) designed to promote tumor‐specific targeting. The following sections will provide a comprehensive and detailed examination of these strategies.

### 7.1 Adoptive NK Cell Therapy

Adoptive NK cell therapy represents a rapidly advancing frontier in cancer immunotherapy, offering distinct advantages over T cell‐based approaches. Unlike T cells, NK cells do not require antigen‐specific priming and can exert cytotoxicity (such as IFN‐γ secretion) in an MHC‐unrestricted manner, reducing the likelihood of tumor immune evasion [164]. Moreover, NK cells facilitate the development of antitumor adaptive immune responses [165]. Conventional anticancer treatments, particularly chemotherapy, often impair NK cell numbers and function. Importantly, unlike T cells, adoptive transfer of allogeneic NK cells has been demonstrated to be safe in transplant recipients, as NK cells do not mediate graft‐versus‐host disease (GvHD), highlighting the feasibility and necessity of NK cell‐based adoptive immunotherapy. Early clinical studies from the 2000s revealed encouraging outcomes of NK cell immunotherapy in patients with advanced‐stage leukemia [166]. The past decade has witnessed a surge in NK cell‐based therapeutic studies, positioning adoptive NK cell transfer as an emerging innovation in cancer immunotherapy. The main sources of therapeutic NK cells include autologous NK cells and allogeneic haploidentical NK cells. Allogeneic NK cells are typically sourced from umbilical cord blood (UCB), embryonic stem cells, or cytokine‐induced ML NK cells [11]. Due to the technical challenges associated with isolating, transducing, and expanding primary NK cells, autologous NK cells are not ideal for adoptive therapy or for the generation of chimeric antigen receptor (CAR)‐engineered NK (CAR‐NK) cells. Consequently, in vitro activation or genetic modification of allogeneic NK cells has emerged as a major research focus for adoptive cancer immunotherapy [167] (for more details, see Table 1).

**TABLE 1:** Adoptive NK cell therapy.

Therapeutic target	Mechanism to improve NK cell function	Were there already drugs available	Agent	Phase of development in cancer therapy		
				Recruiting	Active	Completed
Increase immune activity of NK cells	First‐generation CAR‐NK: upregulates activated extracellular NKG2D receptors to boost NK cell metabolism and immune activity [168, 169] Second‐ and third‐generation CAR‐NK: increases glucose uptake in the TME and maintain OXPHOS [170] Fourth‐generation CAR‐NK: Enhances IL‐2 and IL‐15 secretion	Yes	CAR‐NK therapy	Hematologic tumors: NCT06201247 Metastatic tumors: NCT05213195	Hematologic tumors: NCT04623944	Hematologic tumors: NCT03056339
				Solid tumor: NCT06087341		
				Ovarian cancer: NCT05776355		
				Pancreatic ductal adenocarcinoma: NCT06503497		
SLC1A5, SLC3A2/SLC7A5	SLC1A5 and SLC3A2/SLC7A5 are essential channels on the NK cell membrane that regulate amino acid transport. The high expression of these transporters can, on the other hand, activate the downstream mTOR signaling pathway, thereby enhancing the metabolic capacity of NK cells within the tumor microenvironment. This metabolic enhancement supports the improved survival and antitumor activity of NK cells [101].	Yes	CAR‐NK cells	Preclinical studies: NK cells in vivo		

Abbreviations: OXPHOS, oxidative phosphorylation; TME, tumor microenvironment.

Autologous NK cells are derived from the patient's own blood, eliminating the need for immunosuppressive therapy and carrying minimal risk of GvHD. Although these infused cells are capable of expanding in vivo, they often fail to elicit effective antitumor responses. One plausible explanation is the inhibitory signaling triggered by self‐recognition of MHC class I molecules, which restrains autologous NK cell activity. As a result, growing attention has been directed toward allogeneic NK cell therapies as a more promising alternative [171].

UCB provides a rich reservoir of NK cells, constituting up to 30% of lymphocytes—significantly higher than the ∼10% found in peripheral blood [172]. Upon stimulation, UCB‐derived NK cells produce levels of IFN‐γ and TNF comparable to those from peripheral blood NK cells. Their therapeutic potential is currently being evaluated in several clinical trials (NCT01619761, NCT02280525) [173]. In addition, established NK cell lines such as NK‐92, KHYG‐1, and YT cells represent another source of allogeneic NK cells. NK‐92 cells, in particular, have entered clinical evaluation and exhibit efficient in vitro expansion with a doubling time of approximately 24–36 h. As NK‐92 cells lack endogenous CD16 expression, researchers have genetically engineered variants expressing high‐affinity CD16 and endogenous IL‐2 (termed haNK cells) to enhance their antitumor functions. Clinical trials involving haNK cells, combined with PD‐L1 blockade and tumor vaccines, have shown promising results [174]. Stem cell‐derived NK cells offer a scalable alternative and are being actively explored to address limitations in donor availability. Protocols have been developed to expand CD34+ hematopoietic progenitor cells from UCB by over 2000‐fold, yielding >90% functional NK cells. These cells are not only suitable for large‐scale, homogeneous NK production but are also more amenable to genetic engineering than primary NK cells. Induced pluripotent stem cells (iPSCs) also represent a promising source of therapeutic NK cells, with iPSC‐derived NK cells demonstrating superior antitumor activity compared with UCB NK cells in several murine leukemia models [175].

Among the various adoptive NK cell approaches, CAR‐NK cells have emerged as a particularly promising platform following the success of CAR‐T therapy [101]. Structurally, CAR‐NK constructs typically consist of an extracellular antigen‐binding domain (usually an scFv), a transmembrane region, and an intracellular signaling domain—mirroring the design of CAR‐T cells [176]. CAR‐NK cells hold significant advantages, including a better safety profile with minimal risk of cytokine release syndrome or neurotoxicity. Moreover, allogeneic CAR‐NK cells do not induce GvHD, allowing for more diverse cellular sources. In patients with CD19+ B‐cell lymphoma or chronic lymphocytic leukemia, CAR‐NK cells engineered to express anti‐CD19 CAR, IL‐15, and an inducible caspase‐9 safety switch achieved a 64% complete response (CR) rate, with no major adverse events reported. To enhance metabolic fitness, gene‐edited CAR‐NK therapy has the potential to enhance and sustain the glucose metabolism and immune activity of NK cells by reinforcing the activation of their activating receptors [177]. The evolution of CAR‐NK therapy has led to successive improvements in glucose metabolism and immune function, with distinct features characterizing each generation. Besides, CAR‐NK cells were also engineered to increase the expression of amino acid transporters on the NK cell membrane, which not only enhances amino acid metabolism within NK cells but also activates downstream signaling pathways, such as mTORC and c‐Myc, which are crucial for NK cell proliferation, survival, and immune function.

The first generation of CAR‐NK therapy not only supports the metabolism and immune function of NK cells by increasing the expression of activated extracellular NKG2D receptors, but also independently counters the inhibitory effects of the TGF‐β pathway on glucose metabolism [168, 169]. The second and third generations of CAR‐NK cells, derived from ML or adaptive NK cells, incorporate CD28 and 4‐1BB (CD137) costimulatory domains into the CD3ζ signaling domains. This modification enhances glucose uptake in the TME and sustains the OXPHOS energy supply by preserving mitochondrial health, including mitochondrial mass and membrane potential, within the NK cells [170]. The fourth‐generation CAR‐NK cells includes a component that facilitates the knockdow‐n of cytokine‐inducible SH2‐containing protein (CIS) in the intracellular domain. CIS inhibition boosts the secretion and expression of IL‐2 and IL‐15, subsequently enhancing glucose uptake and depletion capacity in CAR‐NK cells. Currently, CAR‐NK therapies are undergoing full‐scale clinical trials (phases I–II) for both hematological and solid tumors. A recent phase I/II trial (NCT03056339) targeting CD19+ hematological tumors demonstrates initial evidence supporting the safety and efficacy of CAR‐NK therapy [178]. However, additional clinical trials with larger sample sizes are necessary to further explore indications and refine specific treatment strategies [179, 180].

However, the limited in vivo persistence and lack of memory formation in NK cells necessitate either large infusion doses or repeated administrations to maintain therapeutic efficacy. Thus, developing scalable, efficient, and cost‐effective strategies for CAR‐NK cell production remains a major challenge [167].

In summary, adoptive NK cell therapy represents a rapidly evolving immunotherapeutic strategy with considerable promise. The advancement of adoptive NK cell therapy faces two major challenges: (1) optimizing the source of therapeutic NK cells for adoptive transfer; and (2) enhancing the in vivo cytotoxicity and persistence of NK cells. While the first issue has been largely addressed, the second remains a key focus for future research. A genetically modified K562 leukemia cell line expressing membrane‐bound IL‐15, IL‐21, and 4‐1BB ligand has demonstrated remarkable efficacy in expanding NK cells—up to 47, 967‐fold within 6 weeks—without inducing senescence or functional exhaustion. Clinical data have supported the safety of using these feeder cells in patients. The maintenance of NK cell activity is critically dependent on cytokines such as IL‐2 and IL‐15, which are essential for enhancing NK cell activation and function [181].

### 7.2 Targeting the TME to Unleash NK Cells

#### 7.2.1 Targeting Hypoxia

As mentioned in the previous sections, hypoxia is also a critical factor in the TME that influences NK cell metabolic reprogramming and contributes to immune dysfunction. Therefore, alleviating hypoxia is one of the key strategies for restoring NK cell metabolic function. Currently, the primary strategy to alleviate hypoxia involves limiting tumor angiogenesis to decrease consumption of oxygen [182] and targeting proangiogenic pathways—particularly vascular endothelial growth factor receptor (VEGFR), fibroblast growth factor receptor (FGFR), platelet‐derived growth factor receptor (PDGFR), and angiopoietins—has emerged as a key therapeutic strategy to mitigate hypoxia and enhance antitumor immunity [183, 184, 185, 186, 187, 188, 189, 190].

Recent clinical studies have shown that Bevacizumab, a VEGF inhibitor, can serve as an adjuvant therapy to inhibit tumor angiogenesis, enhance immune system function, and improve both tumor‐free and overall survival in patients with solid tumors, including NSCLC, HCC, and renal cell carcinoma [191, 192, 193, 194, 195]. Nintedanib (Ofev), a triple tyrosine kinase inhibitor (TKI) primarily targeting the VEGFR, FGFR, and PDGFR families, has been approved by the United States Food and Drug Administration (US FDA) for treating locally advanced, metastatic, or locally recurrent NSCLC [196]. The ability of nintedanib to potentiate the antitumor efficacy of immunotherapy has been demonstrated in mouse tumor models [197]. However, the use of nintedanib as an adjuvant therapy in tumor immunotherapy clinical trials is currently rare, with only one study (NCT02856425) reporting its safety, specific dosage, and preliminary efficacy in combination with pembrolizumab for treating advanced tumors [198]. Lenvatinib, a multitargeted inhibitor of VEGFR, FGFR, PDGFR, cKit, and Ret, has been US FDA‐approved for treating HCC, thyroid cancer, and advanced renal cell carcinoma [199]. When combined with an immunotherapy regimen, lenvatinib has been shown to significantly improve the survival outcomes of patients with advanced solid tumor (including HCC, renal cell carcinoma, and advanced endometrial cancer) [200, 201, 202]. Moreover, other inhibitors targeting angiogenic genes, including sunitinib, apatinib, sorafenib, regorafenib, and thalidomide, have also shown significant angiogenesis inhibition in preclinical and clinical trials, effectively enhancing the antitumor effects of immunotherapy (for more details, see Table 2) [203].

**TABLE 2:** Strategies targeting TME.

Therapeutic target	Mechanism to improve NK cell function	Were there already drugs available	Agent	Phase of development in cancer therapy		
				Recruiting	Active	Completed
Hypoxia	Inhibiting excessive tumor angiogenesis can improve the hypoxic conditions within the TME.	Yes	Bevacizumab (Avastin, VEGF inhibitor) [204] Lenvatinib (Lenvima, multitarget kinase inhibitor) [205] Sunitinib (Sutent, multitarget RTK inhibitor) [206] Nintedanib (Ofev, triple tyrosine kinase inhibitor on VEGFR1/2/3, FGFR1/2/3, and PDGFRa/b) [207]	Gestational trophoblastic neoplasia: NCT04812002 Metastatic cancer: NCT04848337, NCT05208047 Advanced solid tumor: NCT04008797, NCT02856425 Lung cancer: NCT04973293 Ovarian cancer: NCT0478728 Colorectal cancer: NCT06293014 Central nervous system tumors: NCT05081180 Thyroid cancer: NCT04321954	Advanced solid tumor: NCT02734004 Metastatic cancer: NCT01932125 Brain tumor: NCT04074785 Thyroid neoplasms: NCT03573960 Lung cancer: NCT05273554, NCT03377023 Endometrial neoplasms: NCT04865289 Thymus neoplasms: NCT01621568	Malignant solid tumor: NCT05476341, NCT02432274 Metastatic cancer: NCT02713763 Brain cancer: NCT01811498 Gastrointestinal cancers: NCT00793871 Neuroendocrine tumors: NCT03290079 Ovarian cancer: NCT01583322 Breast cancer: NCT00270413 Colorectal cancer: NCT02780700 Kidney cancer: NCT00465179 Leukemia: NCT00022048 Liver cancer: NCT00049322 Thyroid cancer: NCT0132155 Lung cancer: NCT02496585 Biliary tract cancer: NCT02579616 Prostate cancer: NCT02182063 Uterine cervical neoplasms: NCT02009579
Adenosine (ADO)	ADO accumulates in the TME through the consumption of ATP by the exonucleotidase enzymes CD39 and CD73 derived from tumors. By binding to the A2A adenosine receptor on the cell surface, adenosine inhibits NK cell metabolism and immune function. Blocking the function of CD73 and CD39 or reducing A2A receptor expression can partially restore NK cell glucose metabolism.	Yes	CF102 (adenosine receptor A3 agonist) [208] Oleclumab (anti‐CD73 monoclonal antibody) [209] BMS‐986179 (anti‐CD73 monoclonal antibody) [210] CPI‐006 (anti‐CD73 monoclonal antibody) [211] NZV930 (anti‐CD73 monoclonal antibody) [212] AB680 (small‐molecule CD73 inhibitors) [213] LY3475070 (small‐molecule CD73 inhibitors) CPI‐444 (A2aR Antagonist) [214] AB928 (dual A2aR/A2bR Inhibitors) [215] IPH5201 (CD39 antibody) [216] TTX‐030 (CD39 antibody) [217] ARL67156 (CD39/73 dual inhibitor)	Liver cancer: NCT05201404 Pancreatic cancer: NCT04104672 Biliary tract carcinoma: NCT06048133 Pancreatic ductal adenocarcinoma: NCT06048484 Oligometastatic prostate cancer: NCT03821246 Metastatic cancer: NCT05335941 Rectal cancer: NCT05024097 Liposarcoma: NCT05886634 Non‐small‐cell lung cancer: NCT05633667	Metastatic colorectal cancer: NCT04660812 Prostatic neoplasms: NCT04381832 Non‐small‐cell lung cancer: NCT03337698, NCT04262856, NCT03846310 Pancreatic adenocarcinoma: NCT03193190	Liver cancer: NCT02128958 Advanced solid tumors: NCT02503774 Myeloma: NCT04280328 Prostate cancer: NCT05177770 Colorectal cancer: NCT03720678
Lactate	Alleviating the acidic conditions in the TME.	Yes	Sodium bicarbonate [218]	N/A	N/A	Pancreatic carcinoma: NCT01198821
	Inhibiting LDHA can reduce lactate production, thereby alleviating high lactate concentration and low pH in the TME, which negatively impact NK cell metabolism, survival, and immune function.	Yes	Oxalate (substrate‐competitive inhibitors) [219] Quinoline 3‐sulfonamides (cofactor, (NADH)‐competitive inhibitors) [220] Gossypol (cofactor, (NADH)‐competitive inhibitors) [221, 222] N‐hydroxy‐indole (substrate and cofactor dual competitive inhibitors) [223]	Breast cancer: NCT06133088 Lymphoma: NCT05338931	N/A	Laryngeal cancer: NCT01633541 Lung cancer: NCT00773955 Adrenocortical carcinoma: NCT00848016 Prostate cancer: NCT00666666 Lymphoma: NCT00891072 Glioblastoma: NCT00540722 Laryngeal cancer: NCT01633541 SCCHN: NCT01285635 Brain and central nervous system tumors: NCT00390403 Recurrent plasma cell myeloma: NCT02697344
	Reducing lactate concentration in the TME by inhibiting the lactate transport channels in tumor cells.	Yes	AZD3965 (MCT1 inhibitor) [224, 225]	N/A	N/A	Advanced cancers (solid tumor, lymphoma): NCT01791595

Abbreviations: ADO, adenosine; MCT1, monocarboxylate transporter 1; SCCHN, squamous cell carcinoma of the head and neck; TME, tumor microenvironment; VEGF, vascular endothelial growth factor.

Although no clinical studies have yet explored hypoxia‐targeted therapies as adjuncts to NK cell‐based therapy, the availability of US FDA‐approved hypoxia‐targeted agents and their demonstrated efficacy in combination with immunotherapy from clinical trials suggest their potential as a promising adjunctive strategy. Integrating hypoxia‐targeted therapies into NK cell‐based treatment regimens may provide a novel and reliable approach to enhancing therapeutic efficacy. However, further clinical studies are necessary to establish the optimal combination strategies, dosages, and patient populations that will maximize therapeutic outcomes.

#### 7.2.2 Targeting ADO

As mentioned above, the accumulation of ADO accumulates in the TME as a product of by extensive ATP consumption mediated by tumor‐derived exonucleotidases CD39 and CD73. This accumulation can impair glucose metabolism and suppress immune functions in NK cells through binding the A2A ADO receptor on their surface [226]. Targeting the ADO pathway represents a promising strategy to enhance NK cell‐based immunotherapy, with approaches including inhibition of ADO production, blockade of CD39 or CD73 activity, and antagonism of A2AR signaling.

Among these strategies, CD73 inhibition has received significant attention, with oleclumab emerging as one of the most extensively studied agents in clinical trials [227]. This mAb noncompetitively binds to and inhibits the exonuclease activity of CD73 on the surface of tumor cells, demonstrating significant tumor suppression in preclinical mouse models of homozygous tumors. In a phase I clinical trial (NCT02503774) reported in 2023, oleclumab, both as a monotherapy and in combination with durvalumab, is shown to be safe, well tolerated, and capable of restoring immunotherapeutic antitumor activity in patients with advanced malignancies [209]. The phase II clinical trial (NeoCOAST, NCT03794544) conducted by Cascone et al. [228] in patients with resectable lung cancer shows that neoadjuvant immunotherapy with oleclumab in combination with an anti‐PD‐L1 inhibitor significantly improves immunotherapy outcome. The major pathological response (MPR) rate is 22.2% (95% CI, 6.4–47.6) in the oleclumab combination group, compared with 12.5% (95% CI, 2.7–32.4) in the group receiving immunotherapy alone [228]. Subsequent multiplatform immunoassay analysis reveals that the significant enhancement of systemic immune cell activity in the oleclumab combination group is the primary contributor to the increased MPR observed in patients after treatment. These findings are further corroborated by a phase II clinical trial conducted by Besse et al. [229] (NCT03334617), which also demonstrate improved outcomes in patients with advanced NSCLC receiving oleclumab combination immunotherapy. Beyond oleclumab, several other mAbs targeting CD73, including BMS‐986179, CPI‐006, and NZV930, as well as small molecule inhibitors like AB680 and LY3475070, are currently undergoing clinical or preclinical evaluation [226].

Apart from CD73 inhibition, A2AR antagonists have also been extensively studied as another clinically feasible treatment option to counteract ADO‐mediated immunosuppression, which had been proven to promote immune cell infiltration, particularly CD8+ T cells and NK cells, thereby inhibiting tumor growth and metastasis [230, 231, 232]. CPI‐444 is a notable representative of A2aR antagonists, with the first dosing trial conducted by Fong et al. [233] further confirming its immune cell activation properties and clinical potential. Additionally, dual A2AR/A2BR inhibitors such as AB928, CD39‐blocking antibodies (e.g., IPH5201, TTX‐030), the CD39/CD73 dual inhibitor ARL67156, and the A3 ADO receptor agonist CF102 are currently in various stages of clinical development [226]. For a comprehensive overview of ongoing clinical trials and specific drug developments, refer to Table 2.

Collectively, these studies highlight the therapeutic potential of targeting the ADO pathway to overcome immune suppression in the TME. While CD73 inhibitors and A2AR antagonists have demonstrated efficacy in combination with checkpoint inhibitors, optimizing treatment regimens, identifying predictive biomarkers, and addressing potential resistance mechanisms remain critical challenges.

#### 7.2.3 Targeting Lactate and Low pH

Lactic acid and its role in creating a low pH environment is a significant factor in the impaired metabolic function and reduced antitumor activity of NK cells. Therefore, targeting tumor acidity has emerged as a potential strategy to enhance immunotherapy efficacy. Current approaches primarily focus on neutralizing acidity, inhibiting lactate production, and blocking lactate transport.

The simplest strategy to counteract tumor acidity is pH buffering using sodium bicarbonate (NaHCO_3_). The most direct approach to addressing this issue is neutralization using NaHCO_3_. Preclinical studies have shown promising results—Pilon‐Thomas et al. [234] demonstrated that buffering tumor acidity with NaHCO_3_ led to a threefold increase in tumor response rates to immunotherapy. However, a subsequent phase I clinical trial (NCT01198821) found no significant improvement in patient prognosis, with poor adherence due to gastrointestinal discomfort and the unpleasant taste of bicarbonate therapy. Moreover, gastrointestinal side effects and the unpalatable taste of NaHCO_3_ further compromise patient adherence [235]. These findings highlight the need to optimize buffering agents, drug formulations, and patient selection for improved clinical implementation.

An alternative strategy to mitigate lactate accumulation and acidity in the TME is to inhibit lactate production in tumor cells. Under hypoxic conditions, elevated expression of lactate dehydrogenase (LDH) is crucial for sustaining high glycolytic rates in tumor cells. LDH‐mediated redox reactions facilitate the regeneration of NAD+, which is essential for the continuous production of ATP through glycolysis [236]. The forward reaction, which converted pyruvate to lactate, is catalyzed by LDHA, while the reverse reaction, converting lactate to pyruvate, is catalyzed by LDHB. Most LDHA inhibitors currently under development remain in the preclinical validation stage, with some progressing to clinical trials. Based on their mechanisms, these inhibitors can be classified into several categories: substrate (pyruvate)‐competitive inhibitors, cofactor (NADH)‐competitive inhibitors, dual (substrate and cofactor)‐competitive inhibitors, and other inhibitors with less defined mechanisms. Oxamate, a pyruvate analogue, exhibits a higher binding affinity for LDH compared with pyruvate. It inhibits LDH activity by competitively binding to the enzyme's pyruvate‐binding site, forming an inactive complex. Preclinical trials have demonstrated that Oxamate significantly reduces LDH levels and inhibits a broad spectrum of tumor types [237, 238, 239]. Both gossypol and quinoline 3‐sulfonamides inhibit LDHA by competitively binding to the cofactor NADH. Preclinical trials have demonstrated their significant efficacy in reducing tumor growth and inhibiting LDHA activity [220, 240, 241]. Gossypol is currently undergoing clinical trials for the treatment of lung, prostate, and breast cancers (see Table 2). Additionally, N‐hydroxy‐indole has been reported to act as both a substrate and cofactor competitive inhibitor, further inhibiting LDHA activity. Despite their promising preclinical efficacy, LDHA inhibitors face challenges in clinical translation, likely due to metabolic compensation. This highlights the need for complementary therapeutic strategies.

Since lactate can be recycled via LDHB or exported via monocarboxylate transporters (MCTs), inhibiting lactate transport represents the third viable strategy. Among these, MCT1 and MCT4 are the most promising targets [242, 243]. MCT1 inhibitors, such as AZD3965, are currently in phase I/II clinical trials [244]. AZD3965 effectively inhibits MCT1, leading to lactate accumulation within tumor cells, reduced glycolytic intermediates, and decreased levels of ATP and NADPH. This inhibition results in diminished lactate and glycolysis levels [245]. Furthermore, AZD3965 has shown efficacy in inhibiting tumor growth in combination with adriamycin or rituximab in the Raji Burkitt lymphoma model [246]. In contrast, MCT4 inhibitors are still undergoing drug development and preclinical evaluation. In addition, MCT4 and MCT1/4 coinhibitors have demonstrated promising preclinical efficacy in various cancer models, including oral squamous cell carcinoma (OSCC), human head and neck cancer cell lines, U‐87 malignant glioma cells, and ANC‐1 cells. These agents have been shown to inhibit both glycolysis and tumor cell proliferation [247, 248, 249]. A comprehensive review of current research indicates that MCT1/4 inhibitors effectively mitigated lactic acid buildup in the TME caused by tumor glycolysis. Additionally, these inhibitors significantly enhance antitumor therapy efficacy by directly altering tumor energy metabolism and inhibiting cellular growth and proliferation (for more details about clinical trial of this section, see Table 2).

Targeting tumor acidosis via LDHA inhibition and MCT blockade represents a promising strategy to enhance NK cell‐based immunotherapy. However, given the compensatory metabolic adaptations in tumors, future studies should focus on three key areas: (1) identifying biomarkers to stratify patients who would benefit from lactate‐targeting therapies; (2) exploring combination strategies, particularly integrating MCT/LDHA inhibitors with NK cell‐based therapies; (3) optimizing drug formulations to enhance the clinical feasibility of pH‐modulating agents.

### 7.3 Metabolic Modulation of NK Cells for Therapy

In the TME, NK cells face profound metabolic challenges that compromise their cytotoxicity, proliferation, and persistence. Tumor‐induced metabolic reprogramming, hypoxia, nutrient depletion, and immunosuppressive signaling networks collectively impair NK cell functionality. Emerging evidence has illuminated the central role of metabolic signaling pathways—including mTOR, TGF‐β, SREBP, and c‐Myc—in orchestrating NK cell bioenergetics, redox balance, and effector function. Targeting these pathways pharmacologically has thus become a promising strategy to restore NK cell metabolic fitness and potentiate their antitumor activity. Moreover, interventions that modulate amino acid, glucose, and lipid metabolism, or that inhibit tumor‐specific nutrient competition and suppressive enzymes, offer additional opportunities to recalibrate the metabolic landscape of the TME in favor of NK cell immunity. Recent advances in nanotechnology, mAb engineering, and small‐molecule drug design have enabled the development of targeted therapies with improved precision and translational potential. This section comprehensively reviews the current landscape of pharmacological strategies aimed at rewiring NK cell metabolism, highlighting key molecular targets, representative agents, ongoing clinical trials, and remaining translational challenges. Together, these approaches pave the way for next‐generation NK cell‐based immunotherapies with enhanced efficacy and durability across solid and hematologic malignancies.

#### 7.3.1 Pharmacological Strategies Targeting Key Pathways

Current research has identified that tumors and TME can reprogram NK cell metabolism through multiple signaling pathways including mTOR pathway, TGF‐β pathway, SREBP pathway, and c‐Myc pathway. As one signaling always involves in various metabolic processes in NK cells, targeting these pathways therapeutically can modify the overall metabolic state of NK cells, thereby helping to restore their immune activity.

##### 7.3.1.1 Targeting mTOR Pathway

The mammalian target of mTOR is a highly conserved serine/threonine protein kinase, and its associated pathway plays a crucial role in regulating various biological processes, including metabolism, cancer, immune function, and aging [250]. Recent studies have established that mTOR pathway regulates NK cell maturation, proliferation, and immune function by modulating the metabolism of glucose, lipids, and amino acids under normal physiological conditions. However, in TME, factors such as hypoxia and activation of the TGF‐β pathway impair the mTOR signaling pathway in NK cells, subsequently disrupting their normal metabolism and then diminishing vitality and immune activity.

Currently, the most established strategy for targeting the mTOR pathway involve directly inhibiting the tumor's mTOR pathway to reduce its metabolic rate, thereby alleviating the burden on NK cells for substance and energy uptake in the TME. The mTOR inhibitors, including AZD8055, ABI‐009, CC‐223, Rapamycin, and RAD001, have been widely investigated in clinical trials and effectively applied in immunotherapy or adjuvant therapy across various cancers [251, 252] (see Table 1). However, inhibiting the mTOR pathway in tumors and the broader TME can also disrupt NK cell metabolism, leading to suboptimal treatment outcomes. Therefore, the direct activation of the mTOR pathway in NK cells to restore their metabolic function and enhancing their antitumor activity could be a more optimized therapeutic approach. Thus, BPQDs@HSA nanosystem (human serum albumin‐encapsulated black phosphorus quantum dots) was employed and interacts with toll‐like receptors on the NK cell membrane, activating downstream PI3K–Akt–mTOR signaling, which reprograms NK cell metabolism by enhancing glycolysis and promoting OXPHOS, thereby sustaining NK cell viability and immune function. This treatment strategy has been proven to significantly enhance the antitumor activity of NK cells in preclinical ovarian cancer model, demonstrating strong potential for clinical translation in immunotherapy [253].

##### 7.3.1.2 Targeting TGF‐β Pathway

TGF‐β is a key member of the TGF‐β superfamily, which also includes BMPs, and is widely involved in immunosuppressive activities [254]. From a metabolic regulation perspective, the upregulated TGF‐β signaling pathway in the TME not only directly inhibits the glucose metabolism but also further suppresses overall metabolic capacity of NK cells by inhibiting the mTOR, c‐Myc pathway [63]. Currently, therapeutic strategies targeting the TGF‐β pathway can be broadly categorized into two major approaches: (1) blocking TGF‐β receptor signaling on NK cells to prevent downstream immunosuppressive effects, and (2) directly neutralizing TGF‐β ligands to reduce their bioavailability in the TME. Below, we summarize key inhibitors developed for each approach and their clinical progress.

mAbs or small molecule TGF‐β inhibitors are developed to block the TGF‐β receptors on the surface of NK cells, preventing their interaction with TGF‐β and thereby inhibiting downstream suppressive signaling. Galunisertib, a recently developed inhibitor of TGF‐β receptor 1, has demonstrated safety and tolerability in the treatment of refractory advanced tumors in a phase Ib/II clinical trial (NCT02423343) [255]. An ongoing single‐arm phase II study (NCT02688712) suggests that adjuvant chemotherapy with galunisertib may benefit patients with locally advanced rectal cancer, achieving a preliminary CR rate of 32% (12 out of 38) [256]. Vactosertib (TEW‐7197) is another small molecule inhibitor that targets TGF‐β receptor 1 by binding to its ADO‐5′‐triphosphate site, thereby preventing the phosphorylation of Smad2 and Smad3 proteins, which are key mediators of downstream TGF‐β signaling [257]. The therapeutic safety and tolerability of vactosertib are confirmed in a phase I trial in 2020 and further supported by a phase Ib/II trial in 2024, both of which also show significant inhibition of the TGF‐β pathway during treatment [258, 259]. Notably, an ongoing phase II, open‐label, multicenter study (NCT04515979) is investigating vactosertib in combination with Pembrolizumab for patients with PD‐L1‐positive NSCLC. In addition, TGF‐β receptor inhibitors like SB‐431542 and LY364947 have shown significant inhibition of TGF‐β signaling and tumor growth suppression in preclinical models [257, 260]. Simultaneously, recent studies have identified GARP as an endogenous receptor for TGFβ, capable of activating the downstream TGF‐β pathway upon forming a complex with TGF‐β, which subsequently dysregulated cellular metabolism. Karen et al. [62] observed that the use of GARP antibodies not only restores OXPHOS capacity in NK cells but also enhances their IFNγ secretion, indicating that GARP could serve as a novel target for anti‐TGF‐β receptor therapies.

Another promising strategy is targeting and neutralizing members of the TGF‐β family, such as TGF‐β1, TGF‐β2, TGF‐β3, and Activin‐A, thereby inhibiting TGF‐β activity. NIS793, a human mAb targeting TGF‐β, is shown to inhibit the TGF‐β pathway and enhance the expression of immune‐related genes in a recent phase I/Ib, open‐label, multicenter, dose‐escalation study (NCT02947165) involving patients with advanced tumors, both as a standalone treatment and in combination with other therapies [261]. Developed in recent years, SRK‐181 is another human mAb targeting TGF‐β1 that has demonstrated improved antitumor efficacy in combination with anti‐PD1 therapy across multiple cancer mouse models [262]. In addition, a multicenter phase I study (DRAGON, NCT04291079) is currently investigating SRK‐181 as a monotherapy or in combination with PD‐L1 therapy. A 2018 prospective trial (NCT01401062) also examines fresolimumab as an adjuvant treatment in patients with metastatic breast cancer, finding that fresolimumab is well tolerated and has a strong safety profile to improve the immune response [263]. Alternatively, a strategy to block TGF‐β activity involved constructing a TGF‐β trap by replicating the extracellular domain of the TGF‐β receptor. Bintrafusp alfa (formerly GSK‐4045154, M7824, MSB0011359C) and SHR 1701 are notable drugs developed using this method. Moreover, these antibodies are equipped with an anti‐PD‐L1 heavy chain on the opposite end, allowing for dual‐target inhibition [264, 265]. Although bintrafusp alfa, a dual‐targeted mAb against TGF‐β and PD‐L1, has been extensively studied and widely applied in oncology (with clinical trials ranging from phase I to III, covering advanced solid tumors such as lung cancer, advanced cervical cancer, and biliary tract cancer), which has demonstrated safety, controllability, and immune activation capabilities [266, 267, 268, 269, 270, 271, 272]. There is still a lack of reports on bintrafusp alfa dosing regimens that can significantly enhance its antitumor efficacy and improve patient prognosis. Further clinical trials are necessary to investigate optimal dosing strategies and to identify appropriate patient populations (considering factors such as age, tumor size, and pathology). At the same time, three clinical trials reported in 2022 and 2024, targeting recurrent or metastatic cervical cancer (phase I, NCT03774979), advanced solid tumors (phase I, NCT03774979), and unresectable stage III NSCLC (phase II), have demonstrated that adjuvant therapy with SHR 1701 exhibited promising antitumor efficacy and a manageable safety profile [273, 274, 275]. (For a summary of other TGF‐β pathway‐targeted inhibitors and associated clinical trials, see the Table 3.)

**TABLE 3:** Strategies targeting metabolic signaling pathways in TME.

				Phase of development in cancer therapy		
Therapeutic target	Mechanism to improve NK cell function	Were there already drugs available	Agent	Recruiting	Active	Completed
mTOR pathway	Two strategies for targeting the mTOR signaling pathway: Inhibiting the tumor mTOR signaling pathway to reduce resource depletion in the TME; Enhancing NK cell metabolism directly by activating the PI3K–Akt–mTOR signaling pathway via BPQDs@HSA.	Yes	AZD8055 (mTOR inhibitor) [276] ABI‐009 (mTOR inhibitor) CC‐223 (mTOR inhibitor) [277] Rapamycin (mTOR inhibitor) Everolimus (mTOR inhibitor) [278]	Liver cancer: NCT05201404 Solid tumors: NCT05997056 Endometrial cancer: NCT05997017	Solid tumors: NCT05840510	Liver cancer: NCT03591965 Glioma: NCT03463265 Solid tumors: NCT01177397 Bladder cancer: NCT02009332 Hematologic tumors: NCT02031419 Perivascular epithelioid cell tumor: NCT02494570 Neuroendocrine tumors: NCT03670030 Colorectal cancer: NCT03439462 Lung cancer: NCT01545947
		Yes	BPQDs@HSA [253]	Preclinical studies: ovarian cancer model		
TGF‐β pathway	The upregulated TGF‐β signaling pathway in the TME not only directly inhibits the glucose metabolism of NK cells but also further suppresses their overall metabolic capacity by inhibiting the mTOR signaling pathway. Inhibiting the TGF‐β signaling pathway can reverse metabolic dysregulation in NK cells.	Yes	Fresolimumab (TGF‐β neutralizing antibody) [279] Galunisertib (TGF‐β receptor I inhibitor) [256] NIS793 (anti‐TGF‐β antibody) [280] HLX60 (anti‐GARP monoclonal antibody) SB525334 (TGF‐β1 receptor inhibitor) [281] SRK‐181(TGF‐β1 inhibitor) SHR 1701 (anti TGF‐β and PD‐L1 dual inhibitors) LY364947 (TGF‐β receptor I inhibitor) [257] SB‐431542 (TGF‐β receptor I inhibitor) [282] Vactosertib (TEW‐7197, TGF‐β receptor I inhibitor)[258] Bintrafusp alfa (GSK‐4045154, M7824, MSB0011359C, anti TGF‐β and PD‐L1 dual inhibitors) [266]	Metastatic cancer: NCT04235777 Lung cancer: NCT04515979, NCT05005429 Melanoma: NCT05106023 Lymphoma: NCT05896046 Advanced rectal cancer: NCT05300269 Solid tumors: NCT04407741 Cervical cancer: NCT04708470 Stomach neoplasm: NCT04893252 Advanced desmoid tumor: NCT06219733 Myeloproliferative neoplasm: NCT04103645 Osteosarcoma: NCT05588648 Leukemia: NCT05400122 Thymic cancer: NCT04417660 Soft‐tissue sarcoma: NCT04874311	Rectal adenocarcinoma: NCT02688712 Metastatic cancer: NCT04952753, NCT04935359 Prostate cancer: NCT02452008 Glioma: NCT01682187 Advanced solid tumors: NCT04291079 Lung cancer: NCT05061823 Gastric or gastroesophageal junction cancer: NCT04950322 Esthesioneuroblastoma: NCT05012098 Colorectal cancers: NCT04491955	Liver cancer: NCT02906397 Advanced solid tumors: NCT02947165, NCT02423343 Metastatic cancer: NCT05417386, NCT04396886, NCT04835896 Breast cancer: NCT02672475, NCT0140106 Glioma: NCT01220271 Pancreatic neoplasms: NCT05546411 Pleural malignant mesothelioma: NCT01112293 Renal cell carcinoma: NCT00923169 Multiple myeloma: NCT03143985 Desmoid tumor: NCT03802084 Cervical neoplasms: NCT04551950 Cholangiocarcinoma: NCT04066491 Squamous cell carcinoma: NCT04220775 Urothelial cancer: NCT04349280 Lung cancer: NCT03631706, NCT03840902
SREBP pathway	SREBP is a fundamental factor in regulating glycolysis, OXPHOS, lipid metabolism in NK cells. However, SREBP's role in regulating metabolism in NK cells is hindered by numerous inhibitory factors in the TME. Targeting the SREBP signaling pathway, reducing the concentration of SREBP inhibitors in the TME or increasing the concentration of SREBP can partially improve the metabolism levels of NK cells, thus restoring NK cell antitumor function [72, 283, 284, 285, 286, 287].	No	N/A	Preclinical studies: prostate cancer		
Targeting c‐Myc pathway	Inhibiting GSK‐3 or GSK‐3β can prevent MYC degradation, sustaining NK cell amino acid metabolism, which further restores and maintains NK cell antitumor activity [288].	Yes	9‐ING‐41 (GSK‐3β inhibitor) [289, 290] LY2090314 (GSK‐3 inhibitor) [291]	Metastatic pancreatic adenocarcinoma: NCT05077800 Refractory cancer: NCT04239092	Salivary gland carcinoma: NCT05010629 Hematological malignancies: NCT03678883 Pancreatic adenocarcinoma: NCT05239182	Pancreatic adenocarcinoma: NCT01632306 Advanced or metastatic cancer: NCT01287520 Acute leukemia: NCT01214603
	c‐Myc has been proven to be a crucial transcription factor in regulating NK cell glucose metabolism and can be modulated by the nuclear signaling protein IRE1. By exogenously mimicking IRE1 activation within NK cells, c‐Myc levels can be partially restored, thereby improving amino acid metabolism [292].	No	N/A	Preclinical studies: tumor cell lines in vivo		
Targeting TNFα and Nrf2 pathways	Enhancing the TNFα pathway can restore aerobic glycolysis in NK cells.	Yes	BMS‐986156 (glucocorticoid‐induced TNF receptor‐related protein agonist) [293, 294]	N/A	Advanced solid tumors: NCT04021043	Advanced solid tumors: NCT02598960
	The dysfunction of NK cells within the TME is frequently caused by oxidative stress resulting from lipid peroxidation, which impairs their glucose metabolism. Activating the Nrf2 antioxidant pathway can restore NK cell metabolism and functionality, thus enhancing their antitumor efficacy in vivo [295].	Yes	Omaveloxolone (RTA‐408, Nrf2 antioxidant activator)[296, 297] Auranofin (Nrf2 antioxidant activator)[298, 299]	N/A	Ovarian carcinoma: NCT03456700	Solid tumor: NCT02029729 Breast cancer: NCT02142959 Melanoma: NCT02259231 Lung cancer: NCT01737502 Hematologic tumors: NCT01419691
PPAR‐γ	Activation of PPAR‐γ increases lipid metabolism in NK cells, resulting in intracellular lipid accumulation. Therefore, inhibiting PPAR‐γ expression can help improve lipid metabolism disorders in NK cells [83].	No	N/A	Preclinical studies: lymphoma		
IL‐1β pathway	The IL‐1β pathway facilitates the transfer of lipids from lung‐resident mesenchymal cells to NK cells. Inhibiting the IL‐1β pathway can help alleviate lipid metabolism disorders in NK cells within the TME.	Yes	Canakinumab (anti‐IL‐1β monoclonal antibody) [300, 301, 302]	Lung cancer: NCT06038526 Pancreatic cancer: NCT05984602 Hematologic tumors: NCT04239157	Lung cancer: NCT03631199 Metastatic pancreatic ductal adenocarcinoma: NCT04581343	Lung cancer: NCT03447769, NCT03626545 Solid tumors: NCT02900664

Abbreviations: Akt, protein kinase B; c‐Myc, cellular myelocytomatosis; GSK, glycogen synthase kinase; IL, interleukin‐1β; IRE1, inositol‐requiring enzyme 1; mTOR, mechanistic target of rapamycin; Nrf2, nuclear factor erythroid 2‐related factor 2; OXPHOS, oxidative phosphorylation; PI3K, phosphoinositide 3‐kinase; PIP5K1A, phosphatidylinositol 4‐phosphate 5‐kinase type I gamma; PPAR, peroxisome proliferator‐activated receptor; rhIL, recombinant human interleukin; SREBP, sterol regulatory element‐binding proteins; SREBPs, sterol regulatory element‐binding proteins; TGF, transforming growth factor; TME, tumor microenvironment; TNF‐α, tumor necrosis factor‐alpha.

##### 7.3.1.3 Targeting SREBP Pathway

The SREBP pathway plays a crucial role in regulating lipid and glucose metabolism in NK cells, thereby influencing their immune function. Recent studies have highlighted its activation as a key mechanism for restoring metabolic homeostasis and enhancing NK cell cytotoxicity.

Mechanistically, SREBP activation promotes both lipid synthesis and glycolytic flux in NK cells. Li et al. [72] demonstrated that adequate lipid accumulation enhances NK cell cytotoxicity, while MEF2C acts as a critical regulator of SREBP‐mediated lipid metabolism. In MEF2C‐deficient and MEF2C‐haploinsufficient patients with NK cell dysfunction, lipid supplementation therapy successfully restored MEF2C expression, leading to improved NK cell cytotoxicity [72]. Additionally, Assmann et al. [285] reported that SREBP activation enhances glycolysis and OXPHOS by upregulating ATP‐citrate lyase and the mitochondrial citrate transporter Slc25a1, which are key mediators of the malate shuttle. This metabolic rewiring supports ATP synthesis and sustains NK cell function [285].

Despite its therapeutic potential, SREBP activity is often suppressed in the TME. Tumor cells secrete large quantities of oxysterols, particularly 25‐hydroxycholesterol (25HC), which directly inhibits SREBP activation. Additionally, tumor‐associated macrophages overexpress cholesterol 25‐hydroxylase, further converting cholesterol into 25HC and exacerbating SREBP suppression [303, 304, 305]. Given these inhibitory mechanisms, future therapeutic strategies may focus on either blocking oxysterol production or directly activating the SREBP pathway to restore NK cell metabolism. While no clinical trials targeting SREBP activation in NK cells have been reported to date, this pathway represents a promising avenue for enhancing NK cell‐based immunotherapies.

##### 7.3.1.4 Targeting cMyc Pathway

cMyc is a master regulator of NK cell metabolism, controlling amino acid uptake, glucose metabolism, and OXPHOS to sustain their antitumor activity. Studies have shown that its stability is tightly regulated by GSK‐3β, which targets cMyc for degradation. Pharmacological inhibition of GSK‐3β has emerged as a strategy to enhance NK cell function. Mudgapalli et al. [288] and Hsu et al. [289] demonstrated that GSK‐3β inhibitors, such as elraglusib (9‐ING‐41) and LY2090314, stabilize cMyc and enhance NK cell cytotoxicity [288, 289]. These agents have undergone clinical evaluation in both solid and hematologic malignancies (see Table 3). While early‐phase trials suggest promising safety profiles and preliminary efficacy [289, 291], further large‐scale studies are needed to validate their therapeutic potential.

In addition, a novel regulatory mechanism involving the IRE1–XBP1–cMyc axis has been identified. Dong et al. [292] demonstrated that IRE1 activation upregulates cMyc expression via XBP1, leading to increased OXPHOS activity in NK cells. This finding suggests that targeting IRE1 or XBP1 could provide an alternative approach for modulating NK cell metabolism and function.

##### 7.3.1.5 Targeting TNF‐α and Nrf2 Pathways

Apart from TGF‐β pathway, multiple studies have shown that activating the TNF‐α pathway or the Nrf2 antioxidant pathway can also aid NK cells in the TME in restoring some degree of glucose metabolism, with related drugs undergoing clinical testing [293, 295]. BMS‐986156 is a glucocorticoid‐induced TNF receptor‐related protein agonist, specifically an IgG1 mAb, that targets the TNF‐α pathway. A recent phase I/IIa clinical trial (NCT02598960) demonstrates that BMS‐986156, whether administered alone or in combination with immunotherapy, has a manageable safety profile for treating advanced solid tumors. During treatment, BMS‐986156 promotes NK cell proliferation in patients, resulting in an elevated NK cell count [294, 306]. Omaveloxolone (RTA‐408) and Auranofin are potent activators of the Nrf2 antioxidant pathway. Research has demonstrated that Omaveloxolone (RTA‐408) reduces inflammation in various cancer models and inhibits tumor growth by enhancing immune cell infiltration [296, 297]. Raninga and colleagues [299] treat a triple‐negative breast cancer (TNBC) xenograft model with Auranofin, observing that it effectively kills tumor cells by promoting the infiltration of CD8+ T cells in vivo. Building on these findings, both drugs have been extensively tested in clinical trials for antitumor therapies targeting solid tumors, such as lung cancer and breast cancer, and hematologic malignancies [298]. Furthermore, studies have reported that NK cells can restore their glucose metabolism and boost immune activity by activating the CD98‐mediated mTOR‐glycolysis metabolic axis or by inhibiting FBP1. Agents targeting these pathways to restore NK cell glucose metabolism are currently in the development and preclinical trial validation phases (for more details about clinical trial of this section, see Table 3) [61, 307].

##### 7.3.1.6 Targeting IL‐1β and PPAR‐γ Pathway

Recent studies have begun exploring lipid metabolism as a potential therapeutic target in NK cell‐based therapies. Current strategies mainly focus on inhibiting tumor‐driven pathways that promote excessive lipid uptake and metabolism in NK cells, particularly the IL‐1β signaling pathway and the PPAR‐γ pathway.

One key mechanism contributing to excessive lipid metabolism in NK cells is IL‐1β‐mediated lipid transfer. Activation of IL‐1β promotes lipid exchange between lung mesenchymal stromal cells, NK cells, and tumor cells, primarily through exosome‐like vesicles. This lipid transfer leads to the accumulation of LDs in NK cells while simultaneously inhibiting adipose triglyceride lipase activity, ultimately suppressing NK cell immune function and promoting tumor survival. In a preclinical lung metastatic breast cancer model, Gong et al. [162] demonstrated that IL‐1β blockade restores NK cell function and enhances the efficacy of adoptive NK cell immunotherapy. Canakinumab, a monoclonal IL‐1β inhibitor, has been investigated in multiple clinical trials for solid and hematological malignancies as a regimen of adjuvant chemotherapy or immunotherapy. However, phase III trials in NSCLC (CANOPY‐1, NCT03447769; CANOPY‐2, NCT03626545) did not demonstrate significant survival benefits [300]. These clinical trial findings indicate that further research is required to identify appropriate clinical contexts and optimize protocols for IL‐1β pathway inhibitors.

Another emerging approach involves modulating PPAR‐γ, a key transcription factor involved in lipid metabolism. Increased PPAR‐γ expression in NK cells correlates with immune suppression and reduced IFN‐γ secretion. Kobayashi et al. [83] reported that lymphoma patients and corresponding mouse models exhibit NK cell dysfunction associated with upregulated PPAR‐γ signaling. Consequently, developing inhibitors that target PPAR‐γ present a promising approach to enhancing NK cell immune function through the modulation of lipid metabolism.

#### 7.3.2 Other Strategies Targeting NK Cell Metabolism

##### 7.3.2.1 Targeting Amino Acid Metabolism

Amino acid metabolism is fundamental to NK cell function, influencing their proliferation, cytotoxic activity, and persistence within TME. However, tumor cells outcompete immune cells for essential amino acids, leading to metabolic stress that impairs NK cell function and antitumor immunity. Recent therapeutic strategies focus on restoring amino acid availability for NK cells through two main approaches: (1) inhibiting amino acid‐depleting enzymes in tumors and (2) targeting amino acid transporters to enhance NK cell metabolism.

##### 7.3.2.2 Inhibiting Amino Acid‐Depleting Enzymes in Tumors Cells

Directly inhibiting amino acid‐degrading enzymes in tumor cells can restore amino acid availability for NK cells, thereby improving their metabolic function. Numerous amino acid metabolism enzyme inhibitors targeting tumors have demonstrated substantial efficacy in preclinical trials. Currently, clinical trials investigating these drugs as therapies or adjuvant treatments in both solid and hematological tumors are actively ongoing (see Table 3 for details). CB‐839 and JHU083, two potent glutaminase inhibitors, are currently undergoing clinical evaluation for their role in tumor metabolism regulation [308, 309]. In a preclinical study, the combination of JHU083 with an EGFR peptide vaccine (EVax) in homozygous EGFR‐mutant mice leads to enhanced tumor infiltration of CD8+ T cells and CD4+ Th1 cells with an increasing oxidative metabolism and improves tumor suppression compared with EVax monotherapy [310]. Similarly, the combination of CB‐839 with PD‐1 inhibition demonstrates better therapeutic outcomes in Kras‐mutant STK11‐/Lkb1‐deficient mouse models of lung cancer by restoring CD8+ T cell function and improving the TME [311]. CB‐1158, an arginase inhibitor designed to target tumor Arg metabolism, has been demonstrated in several preclinical studies to effectively restore immune cell activity, thereby enhancing tumor cell killing [312, 313, 314]. Additionally, IDO is a key metabolic enzyme that enables tumors to deplete Trp. IDO1 inhibitors, such as epacadostat and navoximod (GDC‐0919, NLG‐919), which target this enzyme, have progressively advanced to clinical trials for cancer therapy. However, the results from numerous clinical trials for solid tumors to date indicate that IDO1 inhibitors, whether used alone or in combination, have not provided significant benefits for patients [315, 316, 317, 318, 319]. Researchers have identified several key considerations for optimizing future clinical trials. First, selecting the appropriate IDO1 expression threshold for patient enrollment is crucial, as this threshold can vary significantly across different tumor types. Second, ensuring sufficient inhibition of Trp metabolism within the TME is essential to prevent the activation of metabolic bypass pathways, which could lead to the development of resistance to IDO1 inhibitors. Third, the selection of the appropriate IDO1 inhibitor should be tailored to the specific Trp metabolic pathways involved in each tumor type [320].

##### 7.3.2.3 Targeting Amino Acid Transporters in Tumor and NK Cells

Targeting amino acid transporters, such as SLC1A5, SLC3A2, and SLC7A5, represents another promising strategy. These transporters are critical for Gln and branched‐chain amino acid (BCAA) uptake, supporting both tumor growth and immune cell function [101]. One approach involves reducing amino acid depletion in the TME by inhibiting these transporters on the surface of tumor cells. Examples of this strategy include IGN523, an anti‐SLC3A2 mAb, and nanvuranlat (also known as JPH203 or KYT‐0353, an SLC7A5 inhibitor). These amino acid transporter inhibitors have been developed and tested in both AML and advanced solid tumors. In preclinical trials, nanvuranlat demonstrates the ability to promote CD8+ T‐cell infiltration by inhibiting tumor amino acid transporter activity and reshaping the TME [321]. IGN523 acts as a multifunctional therapeutic antibody that not only obstructs tumor amino acid translocation but also facilitates NK cell recognition and recruitment by binding to and aggregating the CD98 antigen on the tumor cell membrane. Furthermore, IGN523 has been shown to trigger caspase‐3 and caspase‐7‐mediated apoptosis in tumor cells, while also enhancing lysosomal penetration into tumors by forming a complex with CD98 and anchoring with LAMP‐1 (for more details about clinical trial of this section, see Table 4) [322].

**TABLE 4:** Strategies targeting amino acids metabolism of NK cell.

Therapeutic target	Mechanism to improve NK cell function	Were there already drugs available	Agent	Phase of development in cancer therapy		
				Recruiting	Active	Completed
Glutaminase	Inhibiting glutaminase can decrease glutamine (gln) consumption within tumors, thereby increasing gln availability and supporting glycolytic metabolism in NK cells.	Yes	CB‐839 (glutaminase inhibitor) [323] JHU083 (glutaminase inhibitor) [324]	N/A	Advanced solid tumors: NCT03872427 Hematologic tumors: NCT03798678 Lung cancer: NCT03831932 Colorectal cancer: NCT03263429 Astrocytoma: NCT03528642	Advanced solid tumors: NCT02771626 Hematologic tumors: NCT03047993 Renal cell carcinoma: NCT03428217 Triple negative breast cancer: NCT03057600 Ovarian cancer: NCT03944902 Lung cancer: NCT04265534
Arginase	Inhibiting arginase can reduce arginine (arg) consumption in TME, thereby increasing gln availability and restoring the glycolytic metabolism of NK cells.	Yes	CB‐1158 (arginase inhibitor) [314]	Preclinical studies: multiple mouse models of cancer		
SLC1A5, SLC3A2/SLC7A5	SLC1A5 and SLC3A2/SLC7A5 are also crucial for the metabolism, growth, and proliferation of cancer cells. Inhibiting these transporters can reduce amino acid consumption in TME, thereby increasing amino acid availability.	Yes	IGN523 (anti‐SLC3A2 monoclonal antibody) [325] JPH203(SLC7A5 inhibitor) [326]	N/A	N/A	Acute myeloid leukemia: NCT02040506 Advance solid tumors: UMIN000016546, UMIN000034080
IDO	IDO not only directly inhibits the proliferation and immune function of NK cells but also impairs their amino acid metabolism. Inhibiting the production of IDO can mitigate tumor‐induced tryptophan depletion and partially restore the immune function of NK cells.	Yes	Epacadostat (IDO1 inhibitor) [318] Navoximod (GDC‐0919, NLG‐919, IDO1 inhibitor) [316, 327] Indoximod (NLG‐8189) [328] Linrodostat (BMS‐986205) [329] PF‐06840003 [330] NLG802 KHK2455 [331] SHR9146 (HTI‐1090) [332] MK‐7162 LY3381916 [333] DN1406131	N/A	Renal cell carcinoma: NCT03260894	Ovarian cancer: NCT01042847 Lung cancer: NCT03322540, NCT03322566 Advanced solid tumors: NCT02471846

Abbreviations: Arg, arginine; Gln: glutamine; IDO, indoleamine 2, 3‐dioxygenase; mTOR, mechanistic target of rapamycin; TME, tumor microenvironment.

##### 7.3.2.4 Targeting Glucose Metabolism

To enhance NK cell function in the TME, targeting glucose metabolism is emerging as a promising therapeutic strategy. Several approaches have been explored, including optimizing glucose availability, improving NK cell metabolism, inhibiting inhibitory receptors, and modulating metabolic pathways (for more details, see Table 5).

**TABLE 5:** Strategies targeting glucose metabolism of NK cell.

Therapeutic target	Mechanism to improve NK cell function	Were there already drugs available	Agent	Phase of development in cancer therapy		
				Recruiting	Active	Completed
Inhibiting glycolysis of tumor	Inhibiting tumor glycolysis can elevate glucose levels in TME.	Yes	2‐DG [218]	N/A	N/A	Prostatic cancer: NCT00633087 Advanced solid tumors: NCT00096707
Increase glucose metabolism status of NK cell	NKG2A and KIRs function as inhibitory receptors on the surface of NK cells. Targeting these receptors can partially restore glucose metabolism in NK cells, particularly enhancing OXPHOS.	Yes	S095029 (anti‐NKG2A) IPH2101 (anti‐KIR antibody) [334] Lirilumab (second generation anti‐KIR antibody) [335, 336, 337] Monalizumab (IPH2201, anti‐NKG2A antibody) [338]	Digestive cancer: NCT06116136	Solid tumor: NCT05162755, NCT02671435	Hematologic tumors: NCT00552396, NCT01248455, EUDRACT 2005–005298‐31 Bladder cancer: NCT03532451 Advanced gynecologic malignancies: CCGT‐IND221 Recurrent or metastatic head and neck cancer: NCT02643550
	Enhancing the CD98–mTOR–glycolysis metabolic axis can restore the ability of NK cells to produce cytokines.	Yes	Glucagon‐like peptide‐1 (GLP‐1) [307]	Preclinical studies: people with obesity		
	FBP1 is an essential regulator of glycolysis that, when activated, suppresses the anticancer activity of NK cells during cancer development. FBP1 inhibition can restore NK cell glucose metabolism, thereby promoting their cytotoxic activity.	Yes	MB05032 (gluconeogenic enzyme FBP1 inhibitor) [61, 339]	Preclinical studies: lung cancer, human inflammatory, and neuropathic pain		

Abbreviations: 1 IL, interleukin; 2‐DG, 2‐deoxyglucose; FBP1, fructose‐1, 6‐biphosphatase; KIRs, killer immunoglobulin‐like receptors; mTOR, mechanistic target of rapamycin; Nrf2, nuclear factor erythroid 2‐related factor 2; OXPHOS, oxidative phosphorylation; rhIL, recombinant human interleukin; TME, tumor microenvironment; TNF‐α, tumor necrosis factor‐alpha.

A critical challenge in the TME is the competition for glucose between tumor cells and immune cells, including NK cells. Increasing glucose availability for NK cells can help improve their antitumor activity. One approach is to inhibit tumor‐dependent glycolytic processes, thus reducing glucose consumption by tumor cells [340]. A promising strategy is using 2‐deoxyglucose (2‐DG), a nonmetabolizable glucose analog that inhibits glycolysis in tumor cells, thereby increasing glucose availability for NK cells. However, the use of 2‐DG as an adjuvant therapy remains in the exploratory phase with limited clinical trial data supporting its efficacy in enhancing NK cell function [341]. The clinical application of 2‐DG as an adjuvant therapy for tumor metabolism inhibition remains exploratory, with limited trial data currently available to substantiate its efficacy in augmenting antitumor treatment.

The approach to restoring the glycolytic capacity of NK cells involves blocking the activation of inhibitory receptors, with NKG2A and KIRs being the primary targets. Monalizumab, a humanized anti‐NKG2A antibody, is regarded as a promising first‐generation adjunctive antitumor immunotherapy, aiming to boost the activity of both T cells and NK cells in antitumor responses. The results of an open‐label phase II clinical trial (COAST, NCT03822351) published in 2022 demonstrate that patients with stage III unresectable NSCLC who received monalizumab in combination with durvalumab immunotherapy exhibit superior overall response rates and progression‐free survival compared with those treated with durvalumab monotherapy [338]. The NeoCOAST trial (NCT03794544) extends the applicability of monalizumab to resectable NSCLC treated with neoadjuvant immunotherapy. The trial results confirm not only the safety and tolerability of monalizumab as a neoadjuvant therapy but also demonstrate that patients receiving monalizumab and durvalumab combination therapy exhibit higher MPR rates compared with those receiving durvalumab monotherapy. Two generations of anti‐KIR antibodies, IPH2101 and lirilumab, have been developed to targe KIRs and are currently being explored in oncology therapeutic regimens. IPH2101 entered a phase I clinical trial for hematologic tumors in 2012 (NCT00552396, 2005‐005298‐31), and the results confirmed its safety and tolerability during treatment [334, 342]. Three phase II clinical trials investigating IPH2101 monotherapy in myeloma (NCT00999830, NCT01222286, NCT01248455) have been completed and the results have not yet been disclosed. Lirilumab, a second‐generation anti‐KIR antibody, is studied by Hanna et al. [335] in a phase II trial reported in 2022 (NCT03341936). The trial, which tests lirilumab in combination with nivolumab in patients with head and neck neoplasms, demonstrates promising efficacy, with a 43% pathological response rate and 2‐year disease‐free survival and overall survival rates of 64 and 80%, respectively, among responders [335]. In a phase Ib trial (NCT03532451), Grivas et al. [336] found that neoadjuvant treatment with lirilumab combined with nivolumab in patients with muscle‐invasive bladder cancer who are unsuitable for cisplatin therapy is safe and well tolerated, with efficacy comparable to other neoadjuvant therapies.

### 7.4 Immune Checkpoint Blockade for NK Cells

With the deepening understanding of immune checkpoints in NK cells, increasing attention has been paid not only to classical inhibitory receptors such as KIR and NKG2A/CD94—described in the section 7.3.2.4 *targeting glucose metabolism* of this paper—but also to other well‐established immune checkpoints including LAG‐3, CTLA‐4, and PD‐1, as well as newly identified NK cell inhibitory receptors such as Siglec, TIM‐3, and TIGIT. The inhibitory roles of these molecules in NK cell‐mediated antitumor immunity, along with the development of corresponding checkpoint inhibitors, have been progressively reported and investigated in clinical studies.

IMP321, a soluble recombinant LAG‐3‐Ig fusion protein, has been shown in vitro to promote the secretion of cytokines such as IFN‐γ and TNF‐α by NK cells from both healthy individuals and cancer patients with metastasis, thereby exerting antitumor immune effects [343]. Relatlimab, a novel anti‐LAG‐3 antibody, can restore NK cell immune responses against leukemia cells, and its combination with lenalidomide enhances NK cell cytotoxicity [344]. Similarly, cetuximab and ipilimumab, both mAbs targeting CTLA‐4, can indirectly modulate NK cell cytotoxicity and cytokine secretion. Cetuximab has been shown in vitro to enhance the expression of activation markers, cytokine production, and cytotoxic activity of NK cells in head and neck squamous cell carcinoma [345]. Likewise, ipilimumab promotes NK cell‐mediated cytotoxicity and TNF‐α release in melanoma [346]. In addition, treatment with ipilimumab alters the subset distribution of mature NK cells in melanoma patients, influencing NK–tumor cell interactions [347]. A similar effect was observed when ipilimumab was combined with N‐803 (an IL‐15 superagonist) and cytokine‐induced ML NK cells (CIML NK cells), leading to increased proportions of proliferating NK cells in patients with relapsed/refractory head and neck cancer [348]. Furthermore, the combination of IL‐2 with ipilimumab exerts a synergistic effect, significantly enhancing NK cell‐mediated antitumor responses [349]. Treatment with another anti‐CTLA‐4 antibody, tremelimumab, in malignant mesothelioma patients resulted in a shift of NK cell phenotypes from an abnormal to a more physiologically normal state [350].

PD‐1, one of the most well‐characterized immune checkpoints, has been shown to regulate NK cell activity. Its mAb Avelumab significantly enhances the cytolytic activity of NK cells against TNBC cells in vitro [351]. Anti‐PD‐1 antibodies can be combined with other mAbs targeting various tumor‐associated molecules, synergistically enhancing NK cell‐mediated antitumor responses. For instance, coadministration with histone deacetylase (HDAC) inhibitors further augments NK cell cytotoxicity. Similarly, combination therapy of avelumab with IL‐2 and IL‐15 enhances NK cell cytokine production capacity [352]. Pembrolizumab, another PD‐1‐targeting mAb, has been shown to improve overall survival in NSCLC patients when administered in combination with allogeneic NK cell therapy (NCT02843204) [353]. Investigating the efficacy and safety of PD‐1‐based combination therapies that leverage NK cell immunity across various cancer types represents a promising direction for future clinical research.

Emerging inhibitory receptors on NK cells, such as Siglec, TIM‐3, and TIGIT, have been identified as novel immune checkpoints. In vitro studies have demonstrated that blockade of these receptors enhances NK cell activation, cytokine production, and cytotoxicity against tumor cells [354]. In preclinical models, the combination of anti‐TIM‐3, anti‐PD‐1, and recombinant IL‐21 has been shown to enhance NK cell responses and delay tumor progression in mice lacking MHC class I molecules [355]. AET2010, an anti‐TIGIT mAb, has been reported to potentiate NK cell‐mediated antitumor immunity [356]. Moreover, in multiple myeloma, combination therapy with anti‐TIGIT antibody, pomalidomide, and dexamethasone (NCT04150965) leads to enhanced NK cell activation [357]. Similar to anti‐TIM‐3‐based immunotherapy, the coadministration of the anti‐TIGIT antibody ociperlimab with anti‐PD‐1 therapy activates NK cell responses and delays tumor growth [358]. These findings, primarily based on in vitro studies, support the therapeutic potential of immune checkpoint blockade targeting NK cells, particularly in combination with other immunotherapies. However, robust in vivo data remain limited and warrant further investigation.

### 7.5 Cytokine‐Based Therapies

Cytokine‐based therapies were another promising strategies to enhance the cytotoxic effects of NK cells, which involve activating receptors on the surface of NK cells, effectively enhancing metabolism, proliferation, differentiation, and antitumor activity of NK cells [359, 360]. Numerous drug that mimic activating cytokines, such as IL‐2, IL‐15, and IL‐18, have been developed [361]. IL‐2 is the first immunotherapy to receive approval for cancer treatment, and a total of 36 IL‐2‐based compounds have entered clinical trials for oncology, with most of these agents still in the early stages of evaluation [362]. NKTR‐214 (Bempegaldesleukin, Nektar Therapeutics, Bristol Myers Squibb) has the most ongoing clinical trials among pegylated IL‐2‐based compounds in oncology immunotherapy, with 20 registered studies to date [363]. However, clinical development of NKTR‐214 was halted in 2022 after it failed to demonstrate efficacy in combination with nivolumab in two phase III trials. TG4010, another promising IL‐2‐based compound, has demonstrated efficacy in enhancing the effects of chemotherapy in two phase II clinical trials (NCT01383148 and NCT00415818) involving patients with advanced NSCLC. These findings suggest that TG4010 could be considered as an adjuvant therapy to improve immune function in cancer patients when combined with immunotherapy or targeted therapy in the future [364]. IL‐15 emerges as another promising target that could bolster tumor therapy by enhancing NK cell‐mediated immunity. ALT‐803 (N‐803) is a complex formed by the IL‐15 hyper agonist N72D mutant and a dimeric IL‐15 receptor Su/IgG1 Fc fusion protein. Bladder cancer is the tumor in which ALT‐803 has undergone the most extensive clinical testing. A phase Ib/IIb trial by Rosser et al. [365, 366] (QUILT‐2.005, NCT02138734) initially demonstrates that intravesical combination therapy with ALT‐803 for bladder cancer is both safe, without inducing systemic cytokine storms, and effective in achieving long‐term antitumor efficacy. In a subsequent phase II/III trial (QUILT 3.032, NCT03022825), intravesical combination therapy is initiated for Bacillus Calmette‐Guérin‐unresponsive nonmuscle invasive bladder cancer, showing significant efficacy. A total of 72% of patients achieved a CR, among whom 59% sustained their response for at least 12 months. The median duration of CR is 19.2 months (range: 7.6–26.4 months). Beyond its promising efficacy in treating bladder cancer, ALT‐803 has also demonstrated potential in treating advanced lung cancer and other solid tumors. In preclinical trials, Fousek et al. [367] demonstrated that ALT‐803 effectively activates the antitumor activity of NK cells in a SCLC xenograft model with successful tumor elimination. Subsequently, Wrangle et al. [368] and Margolin et al. [369] conducted two phase I clinical trials (NCT01727076 and NCT02523469) in patients with advanced lung cancer and solid tumors, confirming the safety of ALT‐803 as both a monotherapy and in combination immunotherapy, while they also observed enhanced antitumor activity in patient immune cells. A recombinant human IL‐15 conjugated with polyethylene glycol, named NKTR‐255, is developed. Preclinical trials demonstrate that NKTR‐255 has a longer in vivo half‐life and exhibits stronger antitumor effects compared with rhIL‐15 [370, 371]. Currently, NKTR‐255 is undergoing multiple clinical trials in patients with solid and hematological tumors (NCT04136756, NCT04616196, NCT05359211, NCT03233854). Despite IL‐18's role in the metabolism, proliferation, differentiation, and antitumor activity of immune cells, including NK cells, recombinant IL‐18 therapies developed with agonist drugs, similar to other cytokine‐based treatments, have shown limited efficacy against various tumors indicated for immunotherapy [372]. Many tumors produce IL‐18 “decoy receptors, ” named IL‐18 binding proteins (IL‐18BP), that binds IL‐18 with high affinity, preventing it from activating T and NK cells within the TME. Studies have reported a 10‐fold to 100‐fold increase in serum concentrations of IL‐18BP in patients treated with recombinant IL‐18, contributing to the failure of these therapies [373]. Zhou et al. [373] developed ST‐067, an engineered IL‐18 variant (DR‐18) resistant to IL‐18BP binding. In preclinical studies, DR‐18 demonstrates potent antitumor activity both as a monotherapy and in combination with immune checkpoint inhibitors (ICIs) such as anti‐PD‐1 [373]. A phase I/II clinical trial (NCT04787042) is currently underway to assess the safety and preliminary efficacy of ST‐067 in treating advanced solid tumors (for more details, see Table 6).

**TABLE 6:** Cytokine‐based therapies.

Therapeutic target	Mechanism to improve NK cell function	Were there already drugs available	Agent	Phase of development in cancer therapy		
				Recruiting	Active	Completed
Enhance the cytotoxic effects of NK cells	IL‐2: Stimulates NK cell activation and proliferation IL‐15: Enhances NK cell survival and cytotoxicity IL‐18: Promotes NK cell activation	Yes	NKTR‐214 (IL‐2 pathway agonist) [363]	Bladder cancer: NCT02138734 Glioblastoma: NCT06061809	Advanced or metastatic solid tumors: NCT04787042 Bladder cancer: NCT03022825 Lung cancer: NCT05096663	Advanced or metastatic solid tumors: NCT04261439 Hematologic tumors: NCT0157249 Lung cancer: NCT02523469, NCT01383148 Merkel cell carcinoma: NCT02465957 Prostatic neoplasms: NCT00040170
			TG4010 (IL‐2 pathway agonist) [362]			
			Recombinant human interleukin‐15 (rhIL‐15, NKTR‐255) [370, 371]			
			NIZ985 (hetIL‐15) [374]			
			ALT‐803 (N‐803, IL‐15 super agonist) [369]			
			ST‐067 (IL‐18 pathway agonist)			

Abbreviation: IL, interleukin.

### 7.6 NK Cell Engagers

Immune cell engagers represent a novel class of bioengineered molecules that utilize multivalent antibody structures to bridge tumor cells and effector immune cells, facilitating the formation of immunological synapses and thereby enhancing immune cell‐mediated cytotoxic responses. NKCEs, in particular, have shown great promise as a next‐generation immunotherapeutic strategy [7].

#### 7.6.1 CD16‐Based NKCEs

NK cells mediate antibody‐dependent cellular cytotoxicity primarily through CD16a, a low‐affinity Fcγ receptor that enables the recognition and lysis of target cells opsonized with IgG1 and IgG3 antibodies. To enhance CD16‐mediated antitumor responses, several strategies have been developed to overcome the limitations associated with CD16 polymorphism and receptor affinity. These approaches include glycoengineering of antibodies to increase their binding affinity for CD16a and the development of Fv fragments specifically targeting CD16 [375]. Promising CD16‐based NKCEs, such as AFM13, AFM24, DF1001, GTB‐3550, and RO7297089, have demonstrated encouraging potential in tumor immunotherapy [7, 375]. In addition to GTB‐3550, second‐ and third‐generation agents—GTB‐3650 and GTB‐5550—have been developed. GTB‐3650 exhibited enhanced NK cell activation and proliferation in mouse models [376], whereas GTB‐5550 demonstrated the ability to stimulate NK cell expansion in various solid tumors by locally delivering IL‐15 in vitro [377]. However, their therapeutic efficacy may be compromised by the downregulation of CD16 expression in the immunosuppressive TME, which remains a significant obstacle to fully unleashing NK cell cytotoxicity in vivo [378].

#### 7.6.2 NKG2D‐Based NKCEs

NKG2D is a cell surface activating receptor broadly expressed on all human and murine NK cells, as well as on all human CD8⁺ T cells. Preclinical studies have demonstrated that NKCEs targeting NKG2D in combination with multiple myeloma‐associated antigens exhibit promising therapeutic potential. Moreover, bispecific NKCEs and trispecific NKCEs directed against different tumor‐associated antigens (TAAs) have also shown potent antitumor activity in preclinical models [379, 380]. In addition, fusion molecules combining NKG2DLs with tumor‐targeting antigen fragments have been developed, further supporting the feasibility of leveraging NKG2D engagement for effective cancer immunotherapy. NKCEs targeting NKG2D exhibit weaker NK cell activation compared with those targeting CD16. However, they may serve as complementary or alternative therapies when CD16 expression on NK cells is reduced in the TME. Similarly, in the complex TME, NKG2D on NK cells may undergo internalization, degradation, or shedding. This highlights the necessity of developing NKCEs with dual targeting of CD16 and NKG2D. Among these, TriNKET NKCEs have been designed to achieve such dual targeting and have shown promising therapeutic efficacy against AML [380].

#### 7.6.3 NKCEs Based on NCRs

This class of NKCEs primarily exerts its antitumor activity by targeting NCRs, such as NKp46 and NKp30, which are preferentially expressed on NK cells. Unlike CD16a and NKG2D, whose expression is often downregulated in cancer patients, NKp46 and NKp30 maintain stable expression levels [379]. Recent studies have shown that NKp46‐targeting constructs, such as NKp46‐ANKET, can effectively control tumor growth and demonstrate therapeutic potential in diseases like AML. Therapeutic agents developed based on the ANKET platform simultaneously target NKp46 and CD16, and have demonstrated superior antitumor efficacy in clinical trials compared with mAbs or NKCEs targeting NKp46 or CD16 alone [381]. Similarly, NKCEs targeting NKp30 have also exhibited promising preclinical efficacy. It has been demonstrated that NKCEs directed at NKp30 are as effective as NKp46‐based NKCEs in promoting NK cell activation [382]. Moreover, NKCEs that simultaneously target EGFR and NKp30 induce significantly stronger NK cell activation than NKCEs targeting NKp30 alone [380].

### 7.7 Rational Combination Strategies

Current clinical studies consistently indicate that monotherapy with NK cell‐based approaches yields limited efficacy against tumors. Consequently, increasing attention has shifted toward combination strategies involving NK cell‐based therapies. Notably, novel approaches employing CAR‐NK cells as the primary therapeutic modality in conjunction with various treatment modalities have been reported in multiple studies, demonstrating potential clinical value. In this work, we summarize and synthesize the reported CAR‐NK cell‐based combination strategies and the current progress in this field [383, 384] (for more details, see Table 7).

**TABLE 7:** Rational combination strategies.

				Phase of development in cancer therapy		
Therapeutic target	Mechanism to improve NK cell function	Were there already drugs available	Agent	Recruiting	Active	Completed
CAR‐NK combination with chemotherapy or radiotherapy	Chemotherapy and radiotherapy can increase tumor‐associated antigen expression on cancer cells, enhancing CAR‐NK recognition and cytotoxicity. Chemotherapy reduces immunosuppressive cells such as Tregs and MDSCs, thereby promoting CAR‐NK proliferation, infiltration, and persistence.	Yes	Cisplatin [385, 386] Paclitaxel [387]	Preclinical studies: NK cells in vivo		
CAR‐NK combination with OVs	OVs selectively lyse tumor cells and enhance CAR‐NK cell infiltration, proliferation, and activation within the TME. OVs release tumor antigens and trigger inflammatory responses, thereby boosting systemic antitumor immunity.	Yes	OV‐IL15C and EGFR CAR‐NK [388, 389]	Preclinical studies: solid tumors		
CAR‐NK combination with immune checkpoint inhibitors (ICIs)	ICIs can reverse TME‐mediated NK cell suppression, restoring NK immune activity.	Yes	Anti‐PD‐L1 monoclonal antibody and PSMA‐directed CAR‐NK‐92 cells [390]	Preclinical studies: prostate cancer, glioblastoma		
CAR‐NK combination with other agents	TKIs and proteasome inhibitors enhance CAR‐NK function. STING agonists activate NK cells. Hyperthermia and PTT improve immune access.	Yes	Regorafenib and EpCAM‐targeted CAR‐NK cells [383] Cabotinib and CAR‐NK‐92 cells [391] Bortezomib and CAR‐NK‐92 cells [392] STING agonist and CAR‐NK‐92 cells [393]	Preclinical studies: lung cancer, orthotopic pancreatic cancer, and other solid tumors		

Abbreviations: ICIs, immune checkpoint inhibitors; EpCAM, epithelial cell adhesion molecule; OVs, oncolytic viruses; PTT, photothermal therapy; STING, stimulator of interferon genes; TME, tumor microenvironment.

#### 7.7.1 CAR‐NK Combination with Chemotherapy or Radiotherapy

In addition to directly killing tumor cells, chemotherapy and radiotherapy can enhance the sensitivity of CAR‐NK cells to recognize tumor cells by upregulating TAAs on the cancer cell surface, thereby promoting their cytotoxic activity [394]. Furthermore, chemotherapy can promote CAR‐NK cell proliferation, infiltration, and persistence within the TME by reducing regulatory T cells (Tregs) and MDSCs [395]. Consequently, combining CAR‐NK cells with chemotherapy can significantly enhance tumor cell killing. Xia et al. [396] reported that radiotherapy combined with CAR‐T or NK cells exerted synergistic cytotoxic effects against pancreatic cancer. In another study, sequential administration of cisplatin followed by CD133–CAR‐NK and CD44–CAR‐NK‐92 cells achieved the most potent cytotoxicity against ovarian cancer stem cell lines compared with NK cells alone [385, 386]. In addition, Siegler et al. [387] encapsulated paclitaxel in synthetic crosslinked multilamellar lipid vesicle nanoparticles, which stably conjugated to ergosterol moieties on the CAR‐NK cell surface via lipid‐lipid interactions. This strategy enabled a stable combinatorial immuno‐chemotherapy effect while reducing systemic toxicity [387].

#### 7.7.2 CAR‐NK Combination with Oncolytic Viruses

Oncolytic viruses (OVs) are multifunctional tumor‐killing agents that not only selectively lyse tumor cells while sparing normal cells, but also directly promote CAR‐NK cell infiltration, proliferation, and activation. In addition, OVs can indirectly enhance antitumor immunity by releasing tumor antigens and triggering inflammatory responses within the TME [395]. In preclinical models, Ma et al. and Li et al. demonstrated that OV‐IL15C in combination with EGFR–CAR‐NK cells synergistically suppressed tumor growth and significantly prolonged survival compared with monotherapy [388, 389]. Given that OV–CAR‐T combinations have already entered phase I clinical trials in solid tumors such as pancreatic cancer and ovarian cancer (NCT05057715, NCT03740256), it is anticipated that the integration of OVs with CAR‐NK therapy will exhibit substantial clinical potential as these strategies continue to be refined.

#### 7.7.3 CAR‐NK Combination with ICIs

As previously mentioned, tumor‐induced remodeling of the TME alters the expression patterns of NK cell receptors and ligands, downregulating activating receptors while upregulating inhibitory receptors. This suppresses NK cell activation and cytotoxicity, may lead to NK cell loss or exhaustion, and ultimately facilitates tumor immune evasion [397]. Therefore, ICIs themselves represent an important strategy to restore NK cell immune activity. In recent years, the remarkable outcomes of ICIs combined with CAR‐T cell therapy in solid tumors have inspired researchers to extend this combinational approach to CAR‐NK therapy [398, 399, 400, 401, 402, 403]. Emerging preclinical evidence has demonstrated that targeting the PD‐1/PD‐L1 axis—by alleviating checkpoint‐mediated immunosuppression while simultaneously leveraging the intrinsic immune activation of CAR‐NK cells—can synergistically enhance antitumor efficacy against refractory solid tumors. For instance, Wang et al. [390] showed that combining an anti‐PD‐L1 mAb with PSMA‐directed CAR‐NK‐92 cells significantly improved antitumor activity in castration‐resistant prostate cancer. Similarly, Strassheimer et al. [404] reported that the combination of CAR‐NK cells and anti‐PD‐1 antibodies promoted cytotoxic T lymphocyte infiltration into the tumor site, triggering in situ immune responses and achieving stronger tumoricidal activity in advanced glioblastoma.

#### 7.7.4 CAR‐NK Combination with Other Agents

Studies have demonstrated that TKIs not only exert direct cytotoxic effects on tumor cells but also modulate the TME to enhance antitumor immunity. In a human colorectal cancer model, Zhang et al. [383] reported that the multikinase inhibitor regorafenib synergized with epithelial cell adhesion molecule (EpCAM)‐targeted CAR‐NK cells to potentiate tumor cell killing. Similarly, Wu et al. [391] found that the multikinase inhibitor cabozantinib upregulated EGFR expression and downregulated membrane PD‐L1 expression in renal cancer cells, thereby augmenting the in vitro cytotoxicity of CAR‐NK‐92 cells against renal carcinoma.

Proteasome inhibitors have been shown to enhance the functional activity of NK cells or increase the susceptibility of tumor cells to NK cell‐mediated cytotoxicity. Based on this rationale, Zhang et al. [392] demonstrated that the combination of bortezomib with CAR‐NK‐92 cells produced superior antitumor efficacy compared with monotherapy in carbonic anhydrase IX‐positive xenograft models.

Stimulator of IFN genes (STING) agonists have been shown to directly activate the STING signaling pathway in NK cells and enhance their antitumor activity by promoting NK cell activation, upregulating activating receptor expression, and downregulating inhibitory receptor expression [405, 406]. In murine tumor‐bearing models, Da et al. [393] demonstrated that the combination of a STING agonist with CAR‐NK‐92 cells effectively inhibited pancreatic cancer proliferation and significantly prolonged survival.

Moreover, mild hyperthermia can facilitate the recruitment of endogenous immune cells by reducing tumor density, enhancing blood perfusion, and promoting antigen release [407]. Based on this rationale, a combinatorial immunotherapeutic strategy integrating photothermal therapy (PTT), low‐dose‐rate brachytherapy, and CAR‐NK cells has achieved promising therapeutic outcomes in murine models of lung cancer, orthotopic pancreatic cancer, and other solid tumors [396, 408, 409].

In summary, although most of these CAR‐NK‐based combination strategies remain at the preclinical stage, they have, to some extent, addressed key challenges faced by single treatment regimen with CAR‐NK cells in solid tumors, including insufficient tumor tissue homing, limited persistence and activity, and tumor immune evasion. Consequently, combination therapies centered on CAR‐NK cells hold considerable promise for clinical translation.

## 8 Conclusion and Prospects

The therapeutic landscape of cancer immunotherapy is rapidly evolving, and NK cells are increasingly recognized as potent effectors in this arena. Unlike T cells, NK cells possess the inherent ability to recognize and eliminate malignant cells without prior sensitization, making them attractive candidates for adoptive cell transfer and engineered immune cell therapies. However, the functional capacity of NK cells is profoundly shaped—and often suppressed—by the hostile conditions of the TME.

The current study delineates how TME‐driven metabolic reprogramming subverts NK cell antitumor function through three interconnected axes: nutrient deprivation, metabolite accumulation, and signaling dysregulation. In noncancerous inflammatory conditions, NK cells balance OXPHOS and glycolysis to sustain cytotoxicity and cytokine production. However, TME stressors—hypoxia, lactic acid, ADO, and tumor‐derived Kyn—force NK cells into a metabolically compromised state. Glucose deprivation (via TGF‐β‐mediated GLUT1 downregulation and HIF‐1α‐driven mitochondrial fragmentation) impairs ATP generation, while lipid peroxidation (ACSL4‐dependent 4‐HNE production) triggers ferroptosis and disrupts membrane integrity. Concurrently, Arg and Gln depletion destabilize cMyc and mTORC1 signaling, silencing effector genes (e.g., IFNG, GZMB) through H3K27me3‐mediated epigenetic repression. These metabolic adaptations, activation or inhibition of signaling pathways and epigenetic regulation collectively result in NK cell exhaustion, enabling tumor immune evasion. Recent advances in targeting metabolic checkpoints (e.g., TGF‐β, CD73/A2AR) and restoring nutrient flux (e.g., Arg supplementation) highlight the therapeutic potential of metabolic modulation to reinvigorate NK cell function. Despite significant advancements in both basic research and clinical studies concerning therapeutic strategies aim at enhancing NK cell metabolism, several limitations remained that should be validated in future research.

From perspective of basic research, first, the effects of different substances in the TME on NK cell metabolism are not yet fully understood. For instance, the accumulation of HIF‐1α induced by hypoxia in the TME can influence tumor metabolism, proliferation, and migration, thereby promoting tumor progression through various pathways. However, its impact on NK cell metabolism and subsequent immune function remains controversial [410]. Future studies are warranted to further elucidate and clarify the dual impact of HIF‐1α on NK cell metabolism and function under both acute and chronic hypoxic conditions, as well as within diverse contexts in TME. Second, research on amino acid metabolism remodeling of NK cells within the TME has predominantly concentrated on glutamate, Trp, and Arg. There is a notable paucity of studies investigating how alterations in the metabolism of other essential and nonessential amino acids impact NK cell immune function. Recent research highlight that BCAAs enhance glycolysis and OXPHOS in CD8+ T cells, leading to improved tumor infiltration and immune effector capacity [411], which could be poised to become a significant future research direction for the metabolic reprogramming of NK cells in TME. Third, the limited research on lipid reprogramming mechanisms in NK cells has resulted in a paucity of clinical strategies aim at enhancing lipid metabolism in NK cells. NK cells in the TME typically exhibit a passive adaptation to the high‐fat environment. Rather than merely addressing lipid accumulation in the TME, focusing on enhancing or accelerating the harmless breakdown of lipids in NK cells to improve lipid tolerance or interrupt detrimental lipid‐induced chain reactions appear to be a more promising direction for clinical research.

From perspective of clinical application, most drugs aimed at enhancing NK cell metabolism through NK cell‐based therapies remain in the phase I–II stages, where dosage, safety, and clinical indications are being explored and evaluated. Although signature of NK cell restoration or activation has been observed with drugs like bintrafusp alfa (TGF‐β pathway inhibitor), epacadostat and navoximod (IDO1 inhibitors), these agents failed to demonstrate significant clinical benefit in terms of objective response rates or overall survival, which may be partly attributed to the lack of validated biomarkers to assess NK cell metabolic fitness prior to treatment. To address this, it is advisable that researchers, during preclinical studies, prioritize the identification of appropriate indications and predictive biomarkers through integrated multiomics approaches including analyses of cellular metabolic function, signaling pathway activity, metabolite profiling, and the expression of metabolism‐related genes and proteins (potential biomarker candidates may include serum LDH, which reflects TME acidity, SLC7A5 expression, an amino acid transporter, and mitochondrial DNA content in circulating NK cells). Furthermore, optimizing the pharmacokinetics of therapeutic agents is crucial, considering the balance between preliminary clinical dosing, side effects, and overall efficacy. Finally, overcoming TME‐driven NK cell exhaustion requires a multidisciplinary pipeline linking mechanistic insights to clinical innovation, researchers should recognize that the suboptimal efficacy of NK cell‐based therapies may stem from multiple rather than single metabolic factors. For instance, combining CAR‐NK therapies with agents that reshape the TME to support NK cell metabolism (such as TGF‐β pathway inhibitors, antiangiogenics agents that alleviate hypoxia, or compounds reducing lactate accumulation) may enhance their immune functionality and potentially improve NK cell therapy outcomes. In addition, the specific selection of combinational agents should be guided by clinical assessment of TME or NK cell metabolism‐related biomarkers, such as serum LDH levels or the expression of transporters like SLC7A5, as previously mentioned. In general, considering the results from current clinical trials, it appears that once issues related to indications, toxicity, and side effects are resolved, metabolism‐targeted therapies become a cornerstone of NK cell‐based immunotherapy. Innovative therapeutic approaches—such as targeting the mTOR and TGF‐β pathways, engineering CAR‐NK cells with enhanced metabolic fitness, and utilizing NKCEs—are paving the way for more effective and durable NK cell‐based immunotherapies. Moreover, integrating metabolic modulators with ICIs, and optimizing in vivo persistence and tumor infiltration, represent key frontiers in translational research.

Epigenetic regulation—including DNA methylation, histone modifications, and noncoding RNAs—has emerged as a critical determinant of NK cell maturation, effector function, and tumor recognition. However, tumor‐induced epigenetic repression of activating receptors, heterogeneity in regulatory patterns, and the complexity of noncoding RNA networks remain major challenges that limit therapeutic efficacy. Future strategies integrating epigenetic reprogramming, RNA‐based engineering, and precision epigenomic profiling—particularly in combination with cytokine stimulation or adoptive NK cell therapy—offer promising avenues to overcome these barriers. Leveraging advances in single‐cell epigenomics and gene‐editing technologies may ultimately enable the stable reprogramming of NK cells, unlocking their full potential as next‐generation cancer immunotherapies. In summary, tumors and TME influence NK cells to undergo metabolic reprogramming, characterized by reduced amino acid and glucose metabolism and increased lipid metabolism, due to their competitive disadvantage for resources and disruption of tumor‐associated signaling pathways. Since metabolic processes in NK cells are crucial for regulating their immune activity, the reprogramming metabolic patterns not only diminish NK cell activity and viability but also impair their immune functions. Therefore, the depletion of antitumor immune activity due to dysfunctional NK cell metabolism in the TME is a major factor in the progression of immune escape and reduced effectiveness of antitumor treatments [340]. Recent advances in TME‐induced metabolic reprogramming of NK cells have highlighted the potential for enhancing or restoring NK cell‐mediated immunity by improving their metabolic status, offering a promising alternative for NK cell‐based antitumor therapies. Therapeutic strategies aimed at improving NK cell metabolism generally follow two main approaches: one is targeting and inhibiting tumor metabolic pathways to reduce resource consumption and harmful by‐products. Another is using gene editing, recombinant cytokines, and blocking immunosuppressive pathways or inhibitory receptors to directly or indirectly restore NK cell immune functions. Recent clinical trials have introduced several drugs and strategies based on these principles, such as mTOR inhibitors and TGF‐β inhibitors, which have shown promising efficacy in treating cancer patients.

The current review not only outlines the directions for basic research and drug development in NK cell‐based therapies but also offers valuable insights for advancing antitumor immunotherapy strategies.

## Author Contributions

Conceptualization: N.L., C.C., and J.Z. Investigation: Y.L, Y.K., C.Z., Y.S., and X.W. Writing–original draft preparation: N.L., C.C., W.Z., J.H., and S.H. Writing–review and editing: N.L., C.C., W.Z., Y.Z., and L.H. Supervision: J.Z. All authors have read and agreed to the published version of the manuscript.

## Conflicts of Interest

The authors declare no conflicts of interest.

## Ethics Statement

The authors have nothing to report.

## Data Availability

The authors have nothing to report.
